# Anti-tau single domain antibodies clear pathological tau and attenuate its toxicity and related functional defects

**DOI:** 10.1038/s41419-024-06927-9

**Published:** 2024-07-30

**Authors:** Sudershana Nair, Yixiang Jiang, Isabella S. Marchal, Elizabeth Chernobelsky, Huai-Wei Huang, Sarah Suh, Ruimin Pan, Xiang-Peng Kong, Hyung Don Ryoo, Einar M. Sigurdsson

**Affiliations:** 1 Department of Neuroscience and Physiology, Neuroscience Institute, New York University Grossman School of Medicine, New York, NY USA; 2 Department of Cell Biology, New York University Grossman School of Medicine, New York, NY USA; 3 Department of Biochemistry and Molecular Pharmacology, New York University Grossman School of Medicine, New York, NY USA; 4 Department of Psychiatry, New York University Grossman School of Medicine, New York, NY USA

**Keywords:** Alzheimer's disease, Neurodegeneration

## Abstract

Tauopathies are a group of neurodegenerative diseases characterized by the presence of tau inclusions. We have developed over fifty anti-tau single-domain antibodies (sdAbs) derived from phage display libraries of a llama immunized with recombinant and pathological tau immunogens. We examined the therapeutic potential of four of these sdAbs in a *Drosophila* tauopathy model following their transgenic expression either in all neurons or neuronal subtypes. Three of these sdAbs showed therapeutic potential in various assays, effectively clearing pathological tau and attenuating or preventing tau-induced phenotypes that typically manifest as defects in neuronal axonal transport, neurodegeneration, functional impairments, and shortened lifespan. Of these three, one sdAb was superior in every assay, which may at least in part be attributed to its tau-binding epitope. These findings support its development as a gene therapy for tauopathies.

## Introduction

The microtubule-associated protein tau (MAPT) is primarily expressed and localized in neurons and to a lesser extent in glial cells [1]. The progressive assembly and deposition of tau plays a key role in tauopathies, including Alzheimer’s disease (AD). The accumulation and aggregation of tau in the neurons leads to the loss of synapses and neurons and eventually, the decline of cognition and sometimes motor function [2–5]. Under physiological conditions, wild-type tau primarily functions to stabilize and promote microtubule assembly and maintenance of the cytoskeleton, evidenced by smaller axons and dendrites in tau knockout mice [6, 7]. Hyperphosphorylation diminishes the ability of tau to interact with microtubules, leading to its aggregation into neurofibrillary tangles, a pathological hallmark of AD [8].

The availability of genetic tools renders *Drosophila melanogaster* a powerful model to dissect cellular processes involved in disease pathogenesis and progression. Several studies have established that the expression of mutant human tau in *Drosophila* recapitulates key pathological features of human tauopathies, including accumulation of hyperphosphorylated tau, neuronal loss, progressive functional deficits, and premature death [9–16].

Immunotherapy is one strategy to target the aggregation of pathological proteins. There are several tau immunotherapies in various phases of clinical trials [17]. Previously, we reported that a single chain variable antibody fragment (scFv) targeting tau suppresses tau-associated lethality in *Drosophila* [18]. Single-domain antibodies (sdAbs), also known as nanobodies or VHHs, have advantages over scFvs, including generally greater affinity for their target and enhanced solubility, resulting in their emergence as alternatives to conventional therapeutic antibodies [19, 20]. SdAbs are naturally found in camelids and are much smaller in size than conventional IgG antibodies (~15 kDa vs ~150 kDa). Their size provides certain advantages such as greater brain entry, binding to hidden epitopes that larger antibodies cannot access, and simpler folding within the cell because of their single unit, which renders them suitable for gene therapy. Their key therapeutic disadvantage over IgGs is their short half-life, which would not be an issue if delivered as a gene therapy, and which provides an advantage for diagnostic imaging. They are also highly amenable to engineering to increase their half-life and enhance their degradation capabilities. We have described in detail the development of our anti-tau sdAbs by immunizing a llama with recombinant tau and pathological tau derived from a human tauopathy brain [21, 22]. We have also reported on the efficacy of two of them in tauopathy culture models and by in vivo microdialysis in a mouse tauopathy model, as well as the suitability of one of them as a diagnostic imaging agent in two different tauopathy mouse models [21, 22].

In this study, we assessed the potential of four anti-tau sdAbs as gene therapy by expressing them transgenically in tauopathy flies. Furthermore, by taking advantage of the ease of genetic manipulation in the flies, we explored their efficacy not only in all neurons but also in different neuronal subtypes for mechanistic insight into their mode of action. Our findings indicate that these sdAbs exhibit varying efficacy in prolonging the lifespan and function of tauopathy flies, which is associated with clearance of pathological tau, when expressed not only pan-neuronally but also in different neuronal subtypes. Hence, these sdAbs have therapeutic potential in tauopathies.

## Materials and methods

### Fly stocks

All *Drosophila* stocks were cultured in standard cornmeal-molasses-yeast medium at 25 °C under 12 h light/12 h dark cycle. To express mutant human tau, we used the previously described UAS-tau^R406W^ line [16]. To study the in vivo efficacy of anti-tau sdAbs, each sdAb transgene with a Myc and His tag at the C-terminus was subcloned into the pUAST-attB plasmid and targeted for insertion into a specific locus on the 2^nd^ chromosome (51C) to generate a stable transgenic line (injection performed by BestGene Inc.). As a negative control for UAS-sdAbs, we generated UAS-EGFP and a control sdAb UAS-Dv^VHH^ [sdAb against Dengue virus, [23]] by injecting them into the same locus as the other sdAbs. Using this approach, we generated flies expressing (1) w; UAS-EGFP, (2) w; UAS-Dv^VHH^, (3) w; UAS-1D9, (4) w; UAS-2B8, (5) w; UAS-1F12, and (6) w; UAS-2F12. The indicated UAS lines were combined with w;; UAS-tau^R406W^/TM3, Sb and then crossed to GAL4 lines such as elav^c155^-GAL4 (BL#458), w; GMR-GAL4 (BL#1104), w; 201Y-GAL4 (BL#4440), UAS-mcd8gfp; Pdf-GAL4 and yw; Pdf-GAL4 (gift from Jae Park, University of Tennessee) lines. For all experiments, only male flies were used.

### Lifespan assay

One hundred newly eclosed flies were collected per genotype and aged in fresh food vials with ten flies in each vial. Surviving flies were counted and transferred to vials with fresh food every other day. A survival curve was generated using percentage survival by consolidating data from three trials for each genotype. To compare survival curves, the log-rank (Mantel-Cox) test was used to determine differences between the genotypes. All statistical analyses were performed using GraphPad Prism version 10.0.2.

### Climbing assay

For the negative geotaxis (climbing) assay, 100 flies from each genotype were initially assayed with some genotypes having fewer flies at the later time points because of reduced survival. Flies from each vial were transferred to an empty vial that was marked every inch from the bottom of the vial prior to the assay. Locomotor activity was assessed by counting the number of flies in the top, middle, and bottom parts of the vial within a 10 s interval after gently tapping flies to the bottom of the vial. The same flies were retested every week until 28 days of age. Each trial was repeated three times, and the climbing ability was calculated as a percentage averaged over three trials per time point.

### RT-qPCR

RNA was extracted from 5-day-old adult fly heads using TRIzol reagent (Invitrogen, Cat # 15596026). cDNA was synthesized via Verso cDNA synthesis kit (Thermo Scientific, Cat # AB1453A) using 1 μg of total RNA. The resultant cDNA was then used 1:10 in the qPCR reaction, which consisted of Power SYBR Green mix (Applied Biosystems, Cat #4367659) and gene-specific primers. Primers are listed in Table 1. The following cycling parameters were used: 95 °C for 5 min, 40 cycles at 95 °C for 10 s, and 60 °C for 45 s, ending with a melting curve. Rp49 was used as the internal reference gene. Three independent biological replicates and three technical replicates for each genotype were assayed.

**Table 1:** Primers used for RT-qPCR.

sdAbs	Primer sequence (5’–3’)
1D9	Forward-CTGAGACTCTCCTGTGCAGC Reverse-GGAGTCGGCATAGTAGGTGC
2B8	Forward-GCGTGAGTTGGTAGCACGTA Reverse-GGTACACCGTGTTCTTGGC
1F12	Forward-CCTGAAACCTGAGGACACGG Reverse-GGCCACTAGTTGAGGAGACG
2F12	Forward-CCGTATCAATGGCATGGGCT Reverse-GCGTTGTCTCTGGAGATGGT
Rp49	Forward-GGTTTCCGGCAAGCTTCAA Reverse-TGTTGTCGATACCCTTGGGC

### Immunostaining

Adult brains were dissected and fixed in 4% paraformaldehyde in 1× phosphate-buffered saline (PBS) for 20 min at room temperature. The fixed tissues were washed with PBS-T (PBS with 0.5% Triton-X100, pH 7.4) followed by blocking with 5% normal goat serum in PBS-T and incubation with the indicated primary antibody overnight. These samples were then washed in PBS-T three times before incubating with a secondary antibody (Alexa Fluor, 1:500, Invitrogen) for 2 h at room temperature. The samples were washed with PBS-T three times followed by a final wash with 1X PBS before mounting in Vectashield (Vector Laboratories). All fluorescent images were obtained using an LSM700 confocal microscope (Zeiss). Multi-labeling images were sequentially scanned and processed with ImageJ and Adobe Photoshop. The following antibodies were used: mouse anti-FasII (1D4, 1:100, Developmental Studies Hybridoma Bank (DSHB), depositor C. Goodman), rat anti-Elav (7E8A10, 1:100, DSHB, depositor Gerald Rubin), mouse anti-PDF (C7, 1:800, DSHB, depositor Justin Blau), rabbit anti-myc (1:1000, Abcam Cat # ab9106) and rabbit anti-GFP (1:500, Invitrogen Cat #A-6455).

### Immunoblotting

Age-matched adult fly heads were collected and homogenized in ice-cold RIPA buffer supplemented with Complete Mini without EDTA protease inhibitors (Roche) and phosphatase inhibitors (Pierce). Protein concentration was measured using the BCA protein assay kit (Pierce) according to the manufacturer’s instructions. Twenty micrograms of total proteins were supplemented with 4X Laemelli buffer and heated at 100 °C for 10 min before loading on 12% Bis-Tris gels. Proteins were transferred to 0.45 μm nitrocellulose membranes (Bio-Rad) that were subsequently blocked in TBS-T buffer (1× Tris-buffered saline with 0.1% Tween 20) with 5% non-fat dry milk for 1 h at room temperature and incubated overnight at 4 °C with the following antibodies: mouse anti-tau (5A6, epitope tau19–46, 1:1000, DSHB, depositor Gail Johnson) and rabbit anti-tau (1:3000, Boster Bio, Cat #A00097-2), mouse anti-phospho-tau (PHF1, epitope p-Ser396,404, 1:1000, gift from Peter Davies and p-Ser202, p-Thr205 (AT8), 1:1000, ThermoFisher, Cat #MN1020), mouse anti-GAPDH (1:3000, SantaCruz, Cat #365062), rat anti-Elav (7E8A10, depositor Gerald Rubin, 1:1000, DSHB) and goat IgG anti-sdAb (VHH) conjugated with peroxidase (1:1000, Jackson ImmunoResearch, Cat #128-035-232). The blots were then incubated with either IRDye 800CW or IRDye 680RD secondary antibodies (1:10,000; LI-COR Biosciences). Bands were quantified using LI-COR Image Studio lite 5.2. To detect sdAbs, the chemiluminescent signal was visualized using a Fuji LAS-4000 and quantified with Image J.

### Rough eye phenotype

The *Drosophila* rough eye phenotype was scored using light microscopy. Four raters were blinded to semi-quantitatively assess the eye for roughness on a 0–4 scale (0 being no rough eye, 1 being least severe, and 4 being most severe). For representative fly eye images, adult flies were dehydrated through a graded series of ethanol solutions, critical point dried, sputter coated, and examined with a scanning electron microscope using the microscopy core facility service at NYU.

### Epitope mapping of the sdAbs against tau peptide libraries using the dot blot assay

The sdAbs 1D9, 1F12, and 2F12 were reacted on a dot blot to assess their binding to a tau peptide library (synthesized by Genscript Inc., Paramus, NJ) to determine their binding epitopes. The library covered all 441 amino acids of the longest isoform of the tau protein, with peptides 1–24 consisting of 25 amino acids and peptide 25 consisting of 9 amino acids. Peptide-peptide overlaps were seven amino acids. For the epitope mapping, peptides were dissolved into a small amount of dimethyl sulfoxide and subsequently diluted into PBS at a concentration of 1 mg/mL, with 5 μg of each peptide dot-blotted onto nitrocellulose membrane and air-dried for 30 min. After blocking the membrane for 1 h with 5% milk in 0.1% Tween-20 in tris-buffered saline (TBS-T), the blots were incubated overnight in a cold room with sdAbs 1D9, 2B8, 1F12, and 2F12 for tau (containing his tag, 0.01 mg/mL) in Superblock (Thermo Fisher Scientific). Following several washes in TBS-T, the blots were then incubated for 1 h at room temperature with anti-6× His tag antibody (1:2000, Abcam, EPR20547) in Superblock, washed with TBS-T, and the signal was detected with IRDye 800CW secondary antibody (1:10,000, LI-COR Biosciences). The bands were quantified by LI-COR Image Studio lite 5.2.

### Isolation of paired helical filament (PHF) tau protein

PHF-enriched tau protein was isolated from a human tauopathy brain as we have described previously [22].

### Biolayer Interferometry (BLI) binding affinity assay

Epitope mapping was followed by BLI (ForteBio Octet RED96) analyses to measure the *K*_**D**_ values for the sdAbs with the most reactive peptides in the dot blot assay. The BLI experiments were carried out in a 96-well black flat bottom plate with a shaking speed set at 1000 rpm, at room temperature. Samples were prepared in 200 μL of 1× PBS assay buffer containing 0.05% Tween-20 at pH 7.4 (PBS-T). To measure the biophysical interaction between the his-tagged sdAbs and two different epitope peptides, we used the Ni-NTA biosensor, which is pre-immobilized with nickel-charged tris-nitrilotriacetic groups to capture his-tagged molecules. Before the BLI analysis, the sensor was hydrated in the assay buffer for 10 min, and a baseline was established in the buffer for 120 s. The Ni-NTA tips were then loaded with his-tagged sdAbs at 5 μg/mL for 120 s, resulting in a specific interaction between the his-tag and nickel ions. Another baseline step was performed in the assay buffer for 120 s, after which the ligand-loaded biosensor tips were dipped into the epitope peptides solutions at different concentrations in PBS-T for 300 s. Finally, dissociation was conducted in the assay buffer for 400 s. A reference biosensor was loaded with the his-tagged sdAb and ran with an assay buffer blank for the association and dissociation steps. The biosensor tips were regenerated by cycling them three times for 5 s each between 10 mM glycine, pH 2, and assay buffer, followed by re-charging for 1 min with 10 mM NiCl_2_.

Data analysis was performed using Data Analysis 11.0 software with reference subtraction using a 1:1 binding model. In a 1:1 bimolecular interaction, both the association and dissociation phases display a time-resolved signal that is described by a single exponential function. Analyte molecules bind at the same rate to every ligand binding site. The association curve follows a characteristic hyperbolic binding profile, with an exponential increase in signal followed by leveling off to a plateau as the binding reaches equilibrium. The dissociation curve follows a single exponential decay with the signal eventually returning to baseline. The complete fitting solution for a 1:1 binding is provided as follows:

Association phase:

$${\rm{y}}={{{R}}}_{\max }\frac{1}{1+\tfrac{{k}_{{\rm{d}}}}{{k}_{{{\rm{a}}}^{\ast}}\, [{\rm{Analyte}}]}}(1-{{\rm{e}}}^{-({k}_{{{\rm{a}}}^{\ast}}\, [{\rm{Analyte}}]+{k}_{{\rm{d}}}){\rm{x}}})$$

Dissociation phase:

$$\begin{array}{c}{\rm{y}}={{\rm{y}}}_{0}{{\rm{e}}}^{-{k}_{{\rm{d}}}({\rm{x}}-{{\rm{x}}}_{0})}\\ {{\rm{y}}}_{0}={{{R}}}_{\max }\frac{1}{1+\tfrac{{k}_{{\rm{d}}}}{{k}_{{{\rm{a}}}^{\ast}}\, [{\rm{Analyte}}]}}(1-{{\rm{e}}}^{-({k}_{{{\rm{a}}}^{\ast}}\, [{\rm{Analyte}}]+{k}_{{\rm{d}}}){{\rm{x}}}_{0}})\end{array}$$

The curve fitting algorithm utilized the Levenberg–Marquardt fitting routine to obtain the best curve fit. In the 1:1 model, the data fits the above equations with *x*, *y*, and Analyte representing time, nm shift, and concentration, respectively. The algorithm determines *R*_max_, *k*_d_, and *k*_a_ values, from which *K*_D_ is calculated by dividing *k*_d_ by *k*_a_ (i.e., *K*_D_ = *k*_d_/*k*_a_).

### Statistics

All data were analyzed with GraphPad Prism version 10.0.2. The lifespan assay was analyzed with the Log-rank test. Protein levels were analyzed by one-way ANOVA followed by Dunnet’s post-hoc test to compare treatment groups with negative control. qRT-PCR data were analyzed by unpaired *t*-test. Axon lengths and mushroom body (MB) morphology were analyzed by one-way ANOVA followed by Dunnet’s post-hoc test for multiple comparisons. Eye phenotype was analyzed by Kruskal–Wallis followed by Dunn’s post-hoc test. The climbing assay was analyzed by two-way ANOVA followed by Tukey’s post-hoc test within each time point.

## Results

### Transgenic expression of sdAbs in neurons

**Fig. 1:**
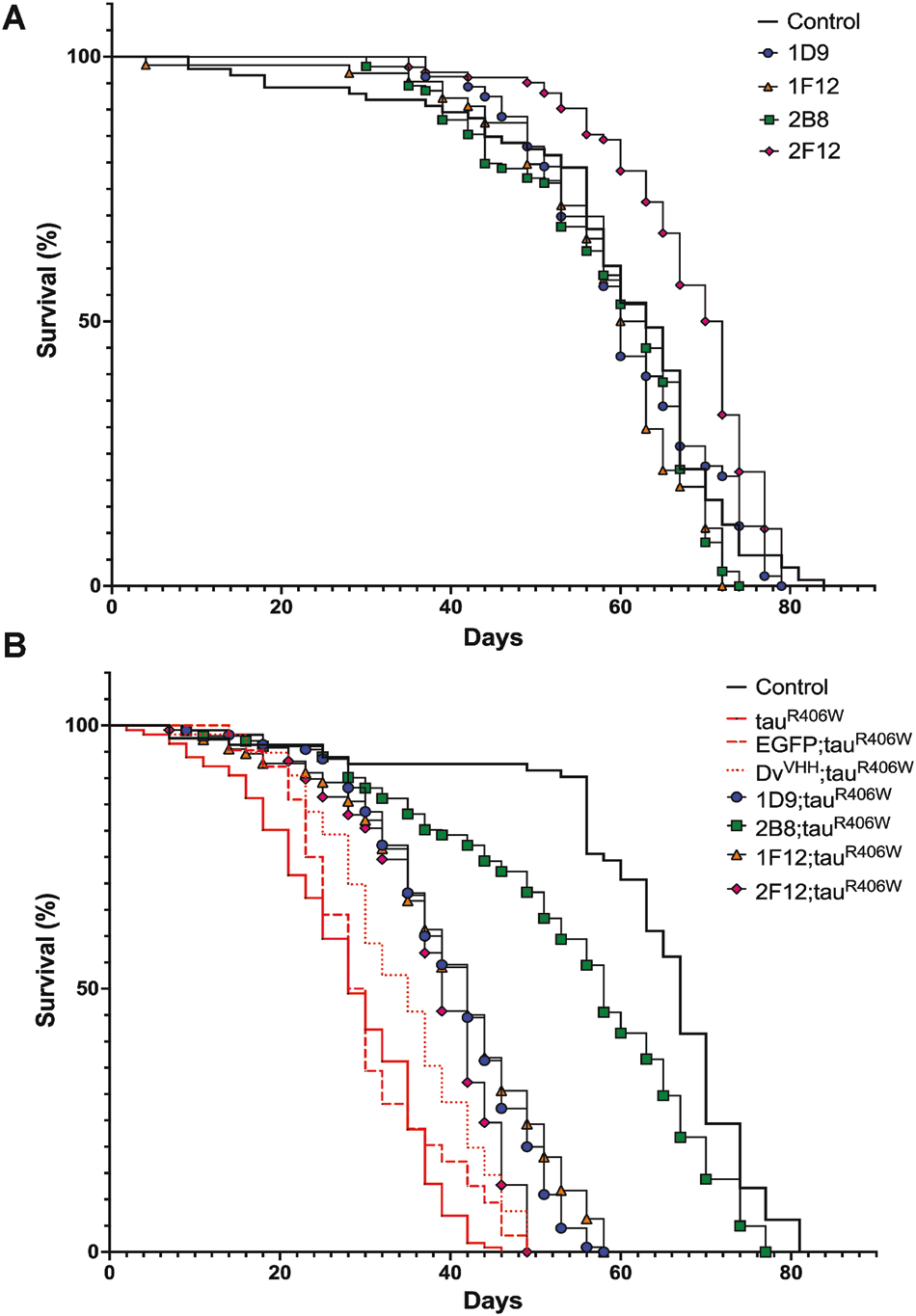
sdAbs suppress tauR406W-induced shortening of adult *Drosophila* lifespan. **A** The effect of expressing the indicated sdAbs alone (without tauR406W) on the adult lifespan. The survival curves are similar between *elav-GAL4/Y* controls and the different sdAbs with no significant difference. Genotypes and the number of flies analyzed per group: 1. *elav-GAL4/Y* (*n* = 86), 2. *elav-GAL4/Y; UAS-1D9/+* (*n* = 109), 3. *elav-GAL4/Y; UAS-2B8/+* (*n* = 53), 4. *elav-GAL4/Y; UAS-1F12/+* (*n* = 64), and 5. *elav-GAL4/Y; UAS-2F12/+* (*n* = 102). **B** The survival curves are very significantly different between *elav-GAL4/Y* controls and tauR406W flies (*p* < 0.0001) with control flies surviving the longest and tauR406W flies surviving the shortest. Both the EGFP and Dv^VHH^ control lines exhibited a shorter lifespan. The sdAb 2F12 did not extend the lifespan (*p* = 0.4110) when compared to the negative controls EGFP and Dv^VHH^, whereas sdAbs 1D9 and 1F12 extended the lifespan by 20 days (Log-rank test, *p* < 0.0001). The anti-tau sdAb 2B8 co-expressed with tauR406W flies live as long as the elav-GAL4 controls (*p* < 0.0001). Genotypes and the number of flies analyzed per group: 1. *elav-GAL4/Y* (*n* = 300), 2. *elav-GAL4/Y;; UAS-tauR406W/+* (*n* = 170), 3. *elav-GAL4/Y; UAS-EGFP/*+*; UAS-tauR406W/+* (*n* = 275), 4. *elav-GAL4/Y; UAS-Dv*^*VHH*^*/+; UAS-tauR406W/+* (*n* = 250), 5. *elav-GAL4/Y; UAS-1D9/*+*; UAS-tauR406W/+* (*n* = 200), 6. *elav-GAL4/Y; UAS-2B8/*+*; UAS-tauR406W/+* (*n* = 300), 7. *elav-GAL4/Y; UAS-1F12/*+*; UAS-tauR406W* (*n* = 200), and 8. *elav-GAL4/Y; UAS-2F12/*+*; UAS-tauR406W* (*n* = 200).

To determine the efficacy of anti-tau sdAbs in *Drosophila*, we generated four different transgenic UAS-sdAbs flies, namely UAS-1D9, UAS-2B8, UAS-1F12, and UAS-2F12. In addition, we generated two different control lines, UAS-EGFP and UAS-Dv^VHH^, an sdAb that recognizes an unrelated epitope of the Dengue virus [23]. The transgenes were all inserted in the same gene locus. We first crossed each of these lines to a pan-neuronal driver elav-GAL4 for their neuronal expression to assess their potential toxicity as examined by gross morphology and lifespan. When expressed pan-neuronally, each of these sdAbs did not appear to have any toxic effects, including on survival (Fig. 1A). We validated the mRNA transcription and protein levels of these sdAbs through q-RT-PCR and western blots, respectively (Supplemental Figs. 1 and 2). We detected robust and comparable mRNA transcription of all the sdAbs using q-RT-PCR. On western blots, we detected comparable amounts of protein for three of the sdAbs, 1D9, 2B8, and 2F12 but we could not detect 1F12. However, 1F12 was clearly detected in whole-mount larval brains (Supplemental Fig. 3), indicating that it is indeed expressed but suggesting that its epitope may not be accessible in western blots as is not uncommon [24].

To further test the therapeutic benefits of these anti-tau sdAbs, we crossed the lines into a *Drosophila* model of tauopathy that features a pan-neuronal expression of tauR406W [16], a mutant form of human tau that underlies a familial form of frontotemporal dementia [25]. Specifically, each sdAb and tauR406W were co-expressed in these flies using the elav-Gal4 driver. Flies expressing tauR406W alone show a severe phenotype with a shorter lifespan of ∼40 days [16], as compared to GAL4 control flies that live close to 80 days (Fig. 1B). The survival curves of the flies that expressed either EGFP or a negative control sdAb (Dv^VHH^) along with tauR406W showed similar shorter lifespans, which were not significantly different from those that expressed tauR406W alone (Fig. 1B). This indicates that both EGFP and Dv^VHH^ do not alter the negative effect of tauR406W on the lifespan of the flies. Among the sdAbs tested, anti-tau sdAb 2B8 was clearly effective in suppressing the shortened lifespan phenotype of tauR406W flies (*p* < 0.0001). These flies lived as long as the elav-GAL4 controls. Two other anti-tau sdAbs, 1D9 and 1F12, modestly suppressed the shortened lifespan of the tauR406W flies, surviving close to 60 days. Although the suppressive effect of these two sdAbs was modest, the differences with the control transgenes were statistically significant (1D9: *p* < 0.0001; 1F12: *p* < 0.0001). The modest suppressive effect of 1F12 may relate to its possibly lower protein levels. In contrast, co-expressing anti-tau sdAb 2F12 did not prolong the lifespan of the tauR406W flies (*p* = 0.4110). These results indicate that the sdAb 2B8 strongly suppresses tau-induced mortality in tauopathy flies, while 1D9 and 1F12 have modest suppressive effects.

### Anti-tau sdAb 2B8 expression prevents climbing defects

**Fig. 2:**
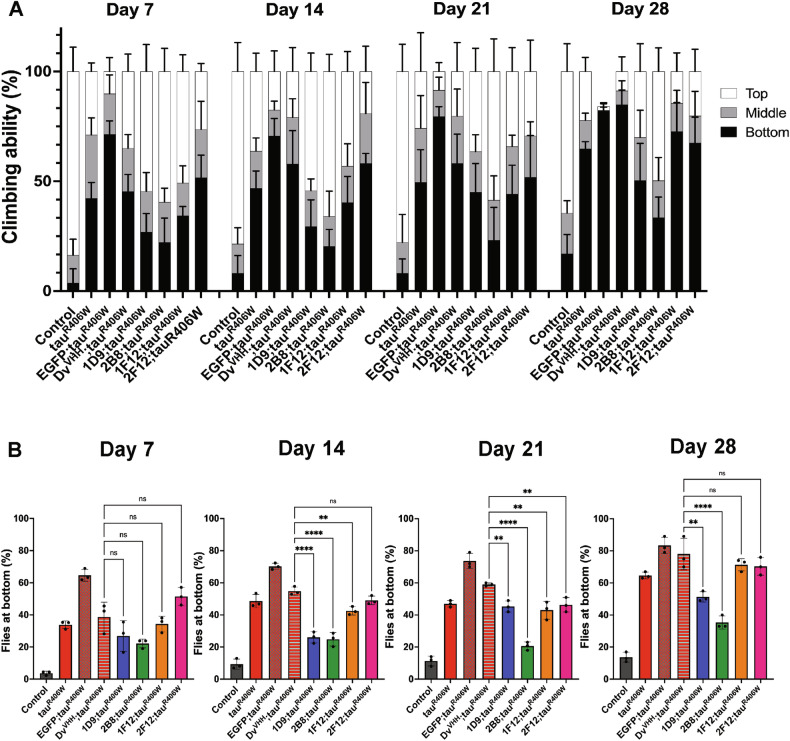
sdAbs prevent locomotion defects observed in tauR406W flies. Climbing ability after tapping of vials was evaluated in flies expressing the indicated transgenes in all neurons using *elav-Gal4*, aged for 7–28 days. More flies reaching to top indicates higher locomotor activity. **A** Data is represented as the average of the percentage of flies at the bottom, middle, and top of the vial from all the trials at the indicated time points for each genotype. The tauR406W flies showed reduced climbing ability on Day 14 and progressively stayed at the bottom of the vial. The anti-tau sdAbs 1D9 and 2B8 rescued climbing defects in flies as old as 28 days. **B** Quantification of the percentage of flies at the bottom of the vial combining all three trials at different time points. Bars represent average ± SD. There were significant genotype, days, and interaction effects for flies at the bottom of the vial (*p* < 0.0001 for all, two-way ANOVA *p ≤ 0.05, **p ≤ 0.01, ***p ≤ 0.001, ****p < 0.0001).

Climbing or negative geotaxis assays are used to assess locomotor functions in adult flies. When tapped to the bottom of a vial, flies display a strong negative geotactic response by rapidly climbing to the top of the vial. Locomotor dysfunction impairs this climbing ability in tauR406W flies [14, 26]. Using this assay as a readout, we asked if the anti-tau sdAbs can prevent locomotor dysfunction in tauR406W flies. The tauR406W flies started to show locomotor dysfunction as early as Day 7 and their climbing ability declined further on days 14, 21, and 28 as compared to the elav-GAL4/Y control flies (Fig. 2A). Flies expressing the negative control EGFP showed increased climbing ability defect. This could be attributed to EGFP itself having a detrimental effect on climbing ability as previously reported [27]. Therefore, we analyzed the climbing ability of sdAb expressing flies to the Dv^VHH^ control, which showed similar climbing ability in younger flies as compared to tauR406W flies (Fig. 2B, Day 7 and 14). To establish if the sdAbs improved the climbing ability of tauR406W flies, we co-expressed in flies each of the sdAbs with tauR406W. In flies as young as 7 days, there was no significant improvement when compared to control sdAb. On Day 14, the anti-tau sdAbs showed statistically significant suppression of the tauR406W-induced climbing defects (1D9: *p* < 0.0001; 2B8 *p* < 0.0001; and 1F12 *p* < 0.0090). The anti-tau sdAb 2F12 did not improve the climbing ability when compared to the control sdAb. However, on day 21, all the sdAbs showed improvement in rescuing the climbing defects (1D9: *p* = 0.0036, 1F12: *p* = 0.0011 and 2F12: *p* = 0.0062), with sdAb 2B8 being the most effective (*p* < 0.0001) as compared to the controls. This could be influenced by sample size variability between the groups due to reduced survival observed in some of them (as per Fig. 1B for a different set of flies). On Day 28, 1D9 (*p* = 0.0036) and 2B8 (*p* < 0.0001) prevented climbing defects in tauR406W flies, whereas sdAbs 1F12 and 2F12 had no effect. Overall, these results indicate that as the flies age, sdAbs rescue to a varying degree the impaired climbing ability of tauR406W flies, with 1D9 and 2B8 being the most effective.

### Anti-tau sdAbs reduce pathological tau protein in tauopathy fly models

**Fig. 3:**
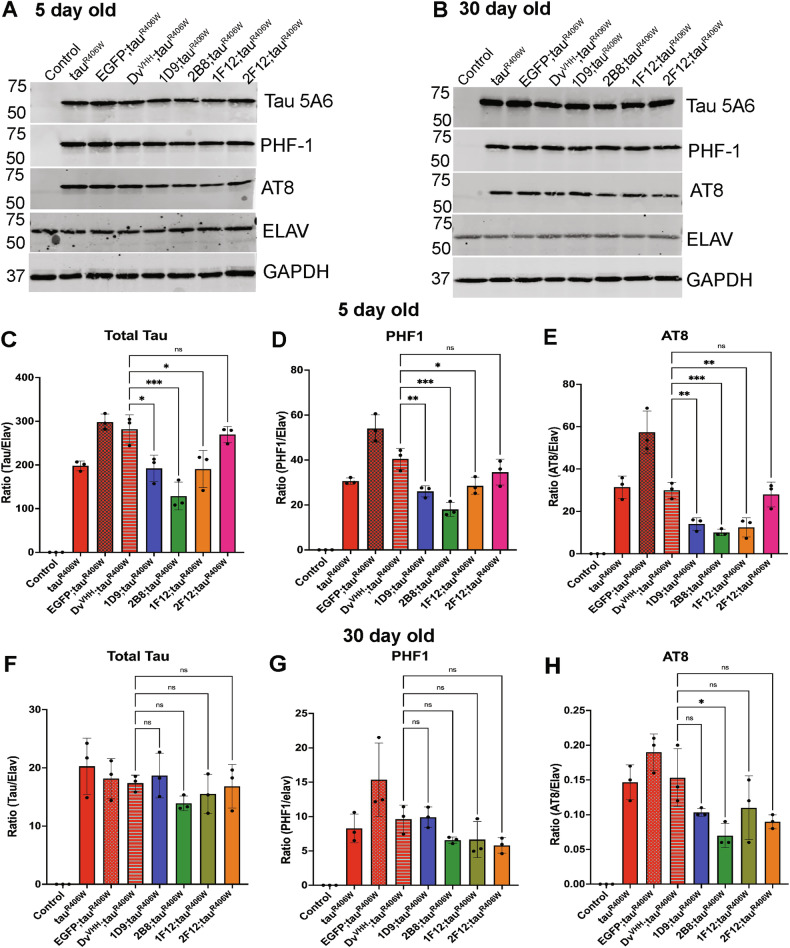
sdAbs clear neuronal tau in young (5-day-old) and older (30-day-old) tauR406W flies. Representative western blots from 5-day-old fly head extracts (**A**) and 30-day-old equivalents (**B**) reacted with total tau antibody 5A6, phospho-tau antibodies PHF-1, and AT8, and antibodies for Elav and GAPDH proteins. **C**–**E** Quantification of tau 5A6 (**C**), PHF1 (**D**), and AT8 (**E**) immunoreactive bands normalized to GAPDH levels and plotted as a ratio to Elav to take into account neuronal loss. GAPDH levels did not differ significantly between the groups. Protein was extracted from 25 fly heads for each biological replicate and each bar represents the average of three blots from three biological replicates with error bars indicating standard deviation (SD). **F**–**H** Quantification of Tau (**F**), PHF1 (**G**), and AT8 (**H**) immunoreactive bands normalized to GAPDH levels and plotted as a ratio to Elav. Bars represent average ± SD (5-day-old tau/Elav: overall *p* = 0.0009, PHF1/Elav: overall *p* = 0.0006, AT8/Elav: overall *p* = 0.0002, 30 day old AT8/Elav: overall *p* = 0.0027, One-way ANOVA, Dunnet’s post-hoc test, *p ≤ 0.05, **p ≤ 0.01, ***p ≤ 0.001). Complete blots for (**A**) and (**B**) are shown in the Supplemental Figs. 4 and 5).

To investigate whether the increased survival of tauopathy flies expressing anti-tau sdAbs was linked to tau clearance, we examined the protein levels of tau and phospho-tau in the flies on Day 5 and Day 30 using three different biological replicates of twenty-five fly heads each (Fig. 3 and Supplemental Figs. 4 and 5). Levels of GAPDH, a housekeeping protein expressed in all cells, did not differ between the groups and were used to normalize the data. We then plotted the ratio of tau to Elav, a protein expressed in neurons, to take potential neuronal loss into account. As expected, control flies had no tau expression whereas tauR406W, EGFP + tauR406W, and Dv^VHH^ + tauR406W showed strong tau expression (Fig. 3). However since EGFP co-expression with tauR406W increased total tau levels more than tauR406W, we compared all the sdAb+tauR406W expressing flies to Dv^VHH^ + tauR406W controls. In the 5-day-old flies, three of the four sdAbs decreased total tau levels (Fig. 3A, C), with 2B8 having the greatest effect (54% reduction, *p* = 0.0001), and 1D9 and 1F12 having a more moderate effect (32% reduction for both, *p* = 0.0209 vs *p* = 0.0190, respectively), whereas 2F12 did not significantly affect tau levels (*p* = 0.0697). The same was true for phospho-tau (Fig. 3A, D–E) as examined using PHF1, which recognizes tau phosphorylated at Ser396/Ser404, and AT8, which binds to phosphorylated Ser 202/Thr205. Here, 2B8 co-expression resulted in the strongest clearance (PHF1: 56% reduction (*p* = 0.0001), AT8: 66% reduction (*p* = 0.0004), with 1D9 and 1F12 having a more moderate effect on PHF1- (1D9: 36% reduction, *p* = 0.0052; 1F12: 30% reduction, *p* = 0.0166) but comparable effect on AT8 immunoreactivity (1D9: 53% reduction, *p* = 0.0022; 1F12: 58% reduction, *p* = 0.0011). Like for tau, 2F12 was ineffective in reducing phospho-tau levels (PHF1: *p* = 0.2926; AT8: *p* = 0.9297). In the 30-day-old flies, none of the sdAbs significantly affected total tau or PHF1 reactive bands, and only 2B8 reduced AT8 bands on the blots (54% reduction, *p* = 0.0187, Fig. 3B, F–H). These results strongly indicate the therapeutic efficacy of three out of the four sdAbs that we examined in the early stages of tau pathology. However, this effect becomes less pronounced at later stages with 2B8 being the most effective sdAb. These results are in line with our survival data, in which the same three sdAbs significantly prolonged the lifespan of tauR406W flies, with 2B8 being the most effective.

### Epitope mapping of the sdAbs against tau peptide libraries and their binding affinity

**Fig. 4:**
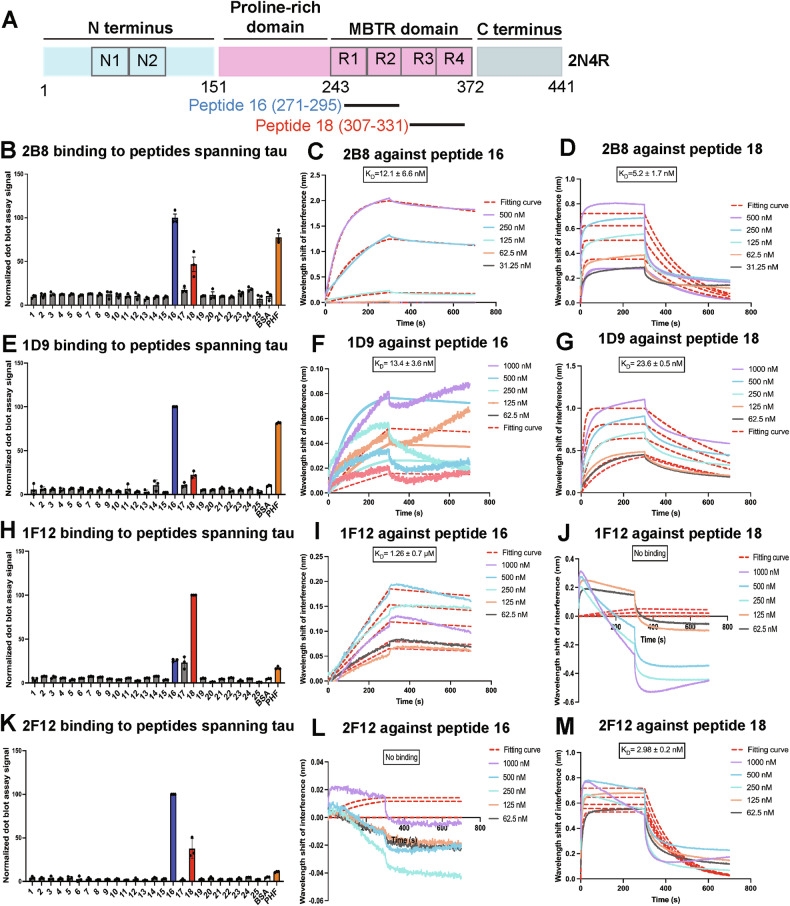
Epitope mapping and binding affinities of anti-tau sdAbs. **A** Domain organization of the largest isoform of tau (2N4R) with 441 amino acids showing peptides 16 and 18. **B** Quantification of a dot blot assay on the binding of sdAb 2B8 to peptides covering all of the 441 amino acids of the tau protein. Each peptide within the library was 25 amino acids except peptide 25, which has 9 amino acids. Each peptide had a 7 amino acid overlap with the following peptide. Only peptides 16 (tau 271–295) and 18 (tau 307–331) displayed strong reactivity with 2B8. Positive control: PHF-enriched tau protein from human tauopathy brain. Negative control: BSA. Each bar shows the mean normalized signal ± SD of three replicates. **C** 2B8 sdAb bound to peptide 16 in solution phase BLI assay with high affinity (*K*_D_ = 12.1 ± 6.6 nM). **D** 2B8 sdAb bound to peptide 18 in the solution phase with high affinity (*K*_D_ = 5.2 ± 1.7 nM). The sdAb is attached to the biosensor, which reacts with different concentrations of peptide 16 and peptide 18. The resulting curves display the shift in wavelength interference in nanometers (nm), which is represented as binding. The curves depict the association and dissociation of sdAbs and tau peptides at different concentrations of tau peptides. The broken line represents the fitting curve used to calculate the *K*_D_ value ± SD which is calculated from three independent experiments. **E** Quantification of a dot blot assay on the binding of sdAb 1D9 to tau peptides showed strong binding to peptide 16 and moderate binding to peptide 18. **F** The sdAb 1D9 bound to peptide 16 with high affinity in the solution phase (*K*_D_ = 13.4 ± 3.6 nM). **G** The binding affinity of sdAb 1D9 to peptide 18 in the solution phase was rather high (*K*_D_ = 23.6 ± 0.5 nM). **H** Quantification of a dot blot assay on the binding of sdAb 1F12 to tau peptides showed strong binding to peptide 18 and moderate binding to peptide 16. **I** The binding affinity of sdAb 1F12 to peptide 16 in the solution phase was moderate (*K*_D_ = 1.26 ± 0.7 μM). **J** The sdAb 1F12 did not bind to peptide 18 in the solution phase. **K** Quantification of a dot blot assay on the binding of sdAb 2F12 to tau peptides showed strong binding to peptide 16 and moderate binding to peptide 18. **L** The sdAb 2F12 did not bind to peptide 16 in the solution phase. **M** 2F12 sdAb bound to peptide 18 in the solution phase with a high affinity (*K*_D_ = 2.98 ± 0.2 nM). See Supplemental Fig. 6 for dot blot assay images and Table 2 for the binding affinity of all sdAbs. This data for 2B8 has been previously reported [22].

To understand why certain sdAbs were effective in prolonging the reduced lifespan of the tauR406W flies, we decided to look at their tau epitopes and binding affinities. We previously reported that sdAbs 2B8 and 1D9 bind to tau tangles and pre-tangles [17, 22]. To determine the specific binding sites of the sdAbs within the tau protein, we mapped their epitopes through a dot blot assay using a peptide library encompassing the longest isoform of the tau protein. The schematic for the 2N4R isoform is shown in Fig. 4A. We have previously reported that 2B8 reacts strongly with peptides 16 (tau 271–295) and 18 (tau 307–331) [22], which are located within the microtubule-binding repeat region of the tau protein [[22], Fig. 4B and Supplemental Fig. 6A]. A follow-up BLI assay showed that 2B8 has a higher affinity for peptide 18 (*K*_D_ = 5.2 ± 1.7 nM) than peptide 16 (*K*_D_ = 12.1 ± 6.6 nM) in the solution phase [[22], Fig. 4C, D]. Epitope mapping of the other three sdAbs revealed solid phase binding to peptides 16 and 18 as well (Fig. 2E, H, and K and Supplemental Fig. 6B, D, and E). Follow-up BLI assay of solution phase binding revealed that 1D9 bound with similar affinity as 2B8 to peptide 16 (*K*_D_ = 13.4 ± 3.6 nM) but with less affinity to peptide 18 (*K*_D_ = 23.6 ± 0.5 nM) (Fig. 4F, G). 1F12 had a substantially lower affinity for peptide 16 in solution (*K*_D_ = 1.26 ± 0.7 μM) and did not bind to peptide 18 (Fig. 4I, J). On the other hand, 2F12 did not bind to peptide 16 but bound with high affinity to peptide 18 in solution (*K*_D_ = 2.98 ± 0.2 nM) (Fig. 4L, M). The binding affinities of each sdAb to peptides 16 and 18 are listed in Table 2 and the sequence of each tau peptide is listed in Table 3. These findings indicate that moderate to high affinity binding to at least peptide 16 in solution is crucial for anti-tau sdAb’s ability to extend the lifespan of tauR406W flies. The most efficacious sdAb, 2B8, binds with the highest affinity to both peptides 16 and 18 in solution. Binding to these peptides in the solid phase does not relate well to efficacy since 2F12 binds well to both peptides in the solid phase but does not extend the lifespan of tauR406W flies.

**Table 2:** Binding affinities of anti-tau sdAbs to peptide 16 and peptide 18 of the 2N4R isoform of recombinant tau comprising of 441 amino acids.

sdAb	Binding affinity to peptide 16	Binding affinity to peptide 18
1D9	*K*_D_ = 13.4 ± 3.6 nM	*K*_D_ = 23.6 ± 0.5 nM
2B8	*K*_D_ = 12.1 ± 6.6 nM	*K*_D_ = 5.2 ± 1.7 nM
1F12	*K*_D_ = 1.26 ± 0.7 μM	No binding
2F12	No binding	*K*_D_ = 2.98 ± 0.2 nM

**Table 3:** Tau peptide library for epitope mapping.

Peptide number	Sequence
Peptide 1 (tau 1–25)	MAEPRQEFEVMEDHAGTYGLGDRKD
Peptide 2 (tau 19–43)	GLGDRKDQGGYTMHQDQEGDTDAGL
Peptide 3 (tau 37–61)	GDTDAGLKESPLQTPTEDGSEEPGS
Peptide 4 (tau 55–79)	GSEEPGSETSDAKSTPTAEDVTAPL
Peptide 5 (tau 73–97)	EDVTAPLVDEGAPGKQAAAQPHTEI
Peptide 6 (tau 91–115)	AQPHTEIPEGTTAEEAGIGDTPSLE
Peptide 7 (tau 109–133)	GDTPSLEDEAAGHVTQARMVSKSKD
Peptide 8 (tau 127–157)	MVSKSKDGTGSDDKKAKGADGKTKI
Peptide 9 (tau 145–169)	ADGKTKIATPRGAAPPGQKGQANAT
Peptide 10 (tau 163–187)	KGQANATRIPAKTPPAPKTPPSSGE
Peptide 11 (tau 181–205)	TPPSSGEPPKSGDRSGYSSPGSPGT
Peptide 12 (tau 199–203)	SPGSPGTPGSRSRTPSLPTPPTREP
Peptide 13 (tau 217–241)	TPPTREPKKVAVVRTPPKSPSSAKS
Peptide 14 (tau 235–259)	SPSSAKSRLQTAPVPMPDLKNVKSK
Peptide 15 (tau 253–277)	LKNVKSKIGSTENLKHQPGGGKVQI
Peptide 16 (tau 271–295)	GGGKVQIINKKLDLSNVQSKCGSKD
Peptide 17 (tau 289–313)	SKCGSKDNIKHVPGGGSVQIVYKPV
Peptide 18 (tau 307–331)	QIVYKPVDLSKVTSKCGSLGNIHHK
Peptide 19 (tau 325–349)	LGNIHHKPGGGQVEVKSEKLDFKDR
Peptide 20 (tau 343–367)	KLDFKDRVQSKIGSLDNITHVPGGG
Peptide 21 (tau 361–385)	THVPGGGNKKIETHKLTFRENAKAK
Peptide 22 (tau 379–403)	RENAKAKTDHGAEIVYKSPVVSGDT
Peptide 23 (tau 397–421)	PVVSGDTSPRHLSNVSSTGSIDMVD
Peptide 24 (tau 415–439)	GSIDMVDSPQLATLADEVSASLAKQ
Peptide 25 (tau 433–441)	SASLAKQGL

To mimic the charge state in the native protein, peptides 2–25 are acetylated on the N-terminus, and peptides 1–24 are amidated on the C-terminus.

### Anti-tau sdAb 2B8 expression prevents tau-induced developmental toxicity

**Fig. 5:**
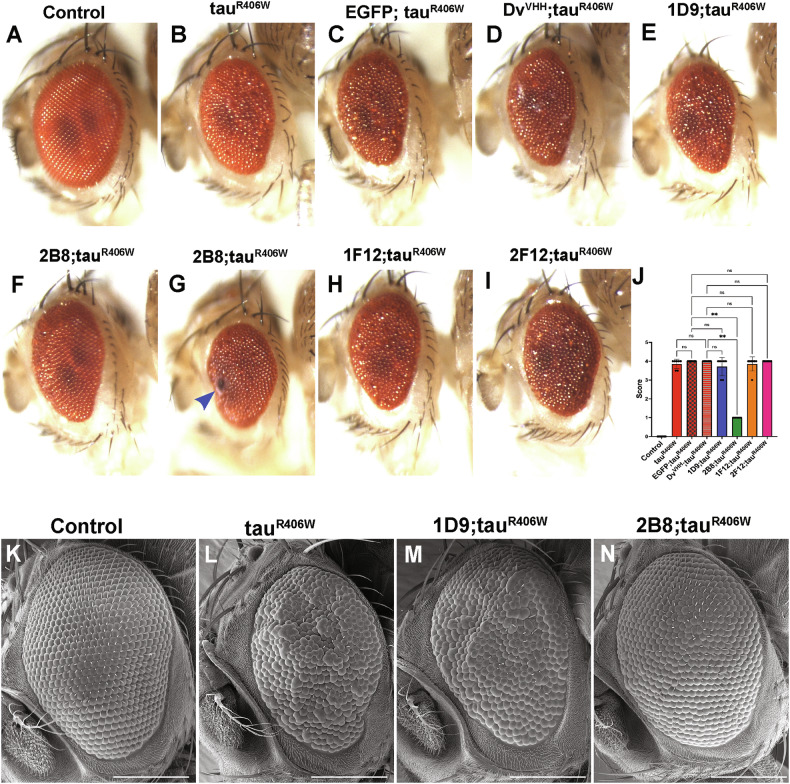
Anti-tau sdAb 2B8 suppresses the rough eye phenotype in the *Drosophila* tauopathy model. Light microscopy images of 5-day-old adult eyes. **A** A control fly eye not expressing tauR406W (genotype, *elav-GAL4/Y*) shows a standard pattern of ommatidia and mechanosensory bristles. **B**–**D** The retinal organization is disrupted in tauR406W-expressing control fly eyes, with ommatidial fusions and missing bristles. Genotypes: **B** *elav-GAL4/Y; UAS-tauR406W/+*. **C** *elav-GAL4/Y; UAS-EGFP/*+*; UAS-tauR406W/+*. **D** *elav-GAL4/Y; UAS-Dv*^*VHH*^*/+; UAS-tauR406W/+*. **E** Expressing anti-tau sdAb 1D9 did not rescue the tauR406W-induced phenotype. Genotype, *elav-GAL4/Y; UAS-1D9/*+*; UAS-tauR406W/+*. **F** However, the ommatidial fusion phenotype was rescued by expressing 2B8. Genotype: *elav-GAL4/Y; UAS-2B8/*+*; UAS-tauR406W/+*. About 5% of the 2B8 flies (*n* = 100) showed dark spots indicated by blue arrow (**G**). Expressing 1F12 (**H**, genotype: *elav-GAL4/Y; UAS-1F12/*+*; UAS-tauR406W*) and 2F12 (**I**, genotype: *elav-GAL4/Y; UAS-2F12/*+*; UAS-tauR406W*) did not suppress the tauR406W phenotype. The semi-quantitative score of the eye images for each genotype is represented in **J** (Kruskal–Wallis, overall *p* < 0.0001, Dunn’s post-hoc test, **p ≤ 0.01). **K**–**N** Representative scanning electron microscope (SEM) images of 5-day-old adult fly eyes of the indicated genotypes: **K** *elav-GAL4/Y*, **L** *elav-GAL4/Y;; UAS-tauR406W/+*, **M** *elav-GAL4/Y; UAS-1D9/*+*; UAS-tauR406W/+*, and **N** *elav-GAL4/Y; UAS-2B8/*+*; UAS-tauR406W/+*. The scale bar represents 100 μm.

The *Drosophila* visual system has been used as an in vivo model to examine how tau toxicity varies with phosphorylation [11, 16]. Pan-neuronal expression of tauR406W disrupts the normal development of photoreceptor neurons, leading to a visible rough eye phenotype in the adult eye morphology with fused ommatidia and missing inter-ommatidial bristles [16]. We examined both light microscopy and scanning electron microscopy images to assess whether the anti-tau sdAbs can suppress this tauR406W-induced developmental toxicity (Fig. 5B, L). We observed that, when co-expressed with tauR406W, our EGFP and Dv^VHH^ sdAb control flies also showed a rough eye phenotype (Fig. 5C, D). The anti-tau sdAbs 1D9 (Fig. 5E, M), 1F12 (Fig. 5H), and 2F12 (Fig. 5I), when co-expressed with tauR406W also produced rough eye phenotypes, indicating that these sdAbs do not efficiently prevent developmental eye toxicity resulting from expressing tauR406W. On the other hand, sdAb 2B8 expression strongly suppressed the eye phenotype caused by tauR406W, with adult flies emerging with very few fused ommatidia (Fig. 5F, N). The quantification of the phenotype score is shown in Fig. 5J. The flies co-expressing 2B8 and tauR406W have several missing mechanosensory bristles, but a significant rescue of the overall retinal organization (*p* = 0.0065). Of note, a very small fraction of the fly eyes expressing both sdAb 2B8 and tauR406W (~5%, *n* = 100) showed melanotic masses (Fig. 5G). It has previously been shown that melanotic masses represent an inflammatory response to abnormal or dying tissue that is too large to be phagocytosed [28]. To address whether the abnormal eye phenotypes shown by tauR406W in the adult stage were associated with defective development during the larval stage, we examined the photoreceptor differentiation process in the third instar larval eye disc using anti-Elav immunostaining. Compared to the control eye disc that showed properly arranged photoreceptors (Supplemental Fig. 7A), tauR406W expression results in disorganized and irregular photoreceptors (Supplemental Fig. 7B). We observed that Dv^VHH^ sdAb control flies, when co-expressed with tauR406W, also show a phenotype similar to tauR406W-expressing eye discs (Supplemental Fig. 7C). The anti-tau sdAbs 1D9 (Supplemental Fig. 7D), 1F12 (Supplemental Fig. 7G), and 2F12 (Supplemental Fig. 7H), when co-expressed with tauR406W, also resulted in disorganized and irregular photoreceptors, indicating that these sdAbs do not efficiently prevent developmental eye toxicity even in larval stages. On the other hand, sdAb 2B8 expression strongly suppressed the tauR406W-associated phenotype with disorganized and irregular photoreceptors (Supplemental Fig. 7E). Of note, a small number of irregular photoreceptors were present in one out of eight larval eye imaginal discs that co-expressed tau together with 2B8 (Supplemental Fig. 7F). Overall, these results indicate that the anti-tau sdAb 2B8 strongly rescued the developmental eye toxicity caused by tauR406W expression in flies.

### Anti-tau sdAb 2B8 expression prevents tau-induced mushroom body (MB) defects

**Fig. 6:**
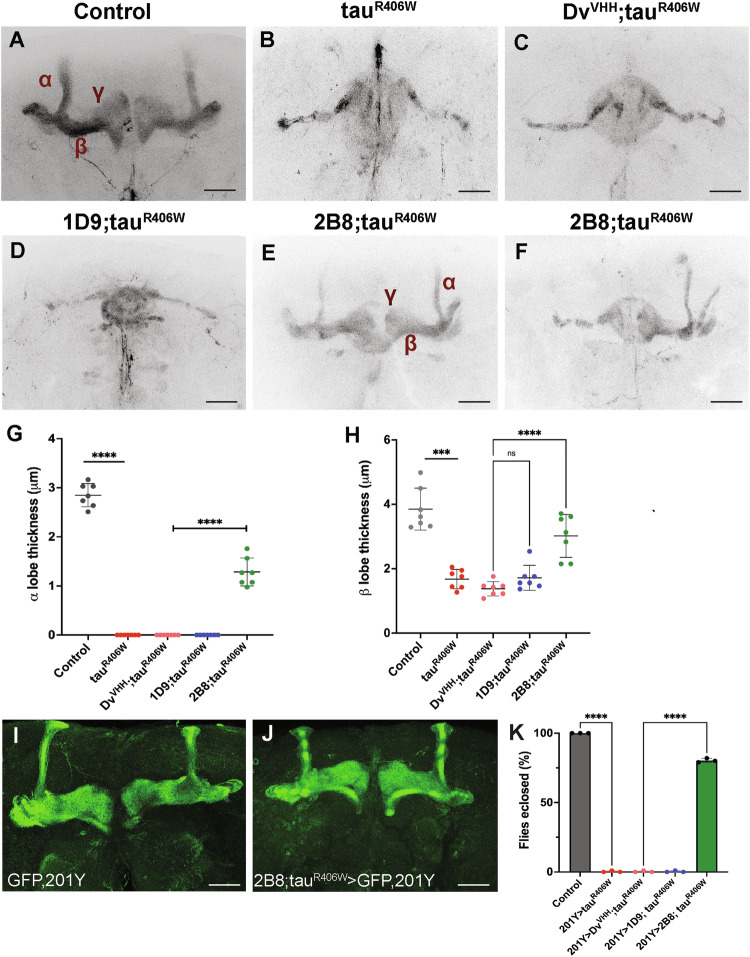
Anti-tau sdAb 2B8 suppresses MB integrity disrupted in tauR406W brains. **A** Adult brains labeled with the anti-FasII antibody on Day 5 show a distinct architecture of the MB in the control *elav-GAL4/Y* flies. **B** By contrast, the α and γ lobes are completely ablated, with thin β lobes remaining, in the flies expressing tauR406W in all neurons (genotype—*elav-GAL4/Y; UAS-tauR406W/+)*. **C** A similar phenotype is observed in the negative controls expressing tauR406W with Dv^VHH^ (genotype—*elav-GAL4/Y; UAS-Dv*^*VHH*^*/+; UAS-tauR406W/+*), or **D** expressing tauR406W with 1D9 (genotype—*elav-GAL4/Y; UAS-1D9/*+*; UAS-tauR406W/+*). **E** 2B8 expression suppressed this tauR406W-associated phenotype with some variability (genotype—*elav-GAL4/Y; UAS-2B8/*+*; UAS-tauR406W/+*), with most of the brains (*n* = 5) showing preservation of the MB structure but thinner α lobe. In other brains (*n* = 3), we observed a wild type like α and β lobe structure but only in one hemisphere (**F**). The scale bar indicates 20 μm. Quantification of the thickness of α lobe (**G**) and thickness of β lobe (**H**) are shown. Error bars show SD. Statistical significance was calculated using one-way ANOVA (overall *p* < 0.0001, Dunnett’s post-hoc test, ***p ≤ 0.001, ****p ≤ 0.0001). Adult brains stained by GFP on Day 5 show MB structure in **I** *UAS-mcd8gfp, 201YGAL4/+* and **J** *UAS-2B8/ UAS-mcd8gfp, 201YGAL4; UAS-tauR406W/+*. Quantification of the percentage of flies eclosed (**K**) is shown and statistical significance was calculated using one-way ANOVA (overall *p* < 0.0001, Dunnett’s post-hoc test, ****p ≤ 0.0001).

Next, we examined the effect of pan-neuronal expression of sdAbs on the *Drosophila* adult MB, a brain region that is particularly vulnerable to tauR406W expression [29]. MB is a bilaterally symmetrical structure with 2500 intrinsic neurons that form the Kenyon cells. The dendrites of these neurons form the calyx whereas the axons fasciculate into the peduncles bifurcating into α/α’, β/ β’, and γ lobes [30–32]. MBs are essential in a wide variety of learning and memory processes including olfactory coding and olfactory learning, as well as memory and decision-making [33–39]. As previously described, Fasciclin II (FasII) labels α/β lobes and weakly labels γ lobes [30]. As compared to the control flies with intact α, β, and γ lobes (Fig. 6A), expressing tauR406W completely ablated α and γ lobes while leaving a very thin β lobe (Fig. 6B). Co-expressing Dv^VHH^ sdAb with tauR406W also resulted in a degeneration phenotype in the MB (Fig. 6C). The anti-tau sdAb 1D9 did not improve the tau-induced degeneration of MBs as compared to flies expressing tauR406W alone (Fig. 6D). On the other hand, co-expressing anti-tau sdAb 2B8 significantly suppressed tauR406W-induced MB degeneration (Fig. 6E). Specifically, most brain samples (*n* = 5) maintained intact α, β, and γ lobes, while some samples (*n* = 2) showed thinner α lobes and β lobes. In other samples (*n* = 3), there were intact α and β lobes with the same width but only on one side of the brain (Fig. 6F). Quantification of the width of the α lobe confirmed a statistically significant suppression of the tauR406W phenotype by 2B8 (Fig. 6G, *p* < 0.0001). While we observed variability in β lobe thickness in the 2B8 phenotype, the difference was still statistically significant as compared to negative controls (Fig. 6H, *p* < 0.0001). These results indicate that anti-tau sdAb 2B8 can prevent tau-induced malformation of the MB when expressed pan-neuronally.

To independently assess the effect of tauR406W in the MB, we followed up on these studies by restricting expression to the MB, which was achieved by the 201Y-GAL4 strain (Fig. 6I). Perhaps reflecting a stronger driver activity, expressing tauR406W using 201Y-GAL4 resulted in pupal lethality with no adult survivors. Similarly, expressing the negative control Dv^VHH^ or anti-tau sdAb 1D9 along with tauR406W also resulted in pupal lethality. Interestingly, however, co-expressing 2B8 and tauR406W completely rescued the lethality and fully preserved the structure of the MB (Fig. 6J). The percentage of eclosed flies is presented in Fig. 6K, revealing a statistically significant difference between sdAb 2B8 and control sdAb (*p* < 0.0001). These results indicate that anti-tau sdAb 2B8 prevents tau mutation-induced lethality caused by targeted expression in the MB.

### Anti-tau sdAb 2B8 expression prevents tau-induced axonal protein distribution defects

**Fig. 7:**
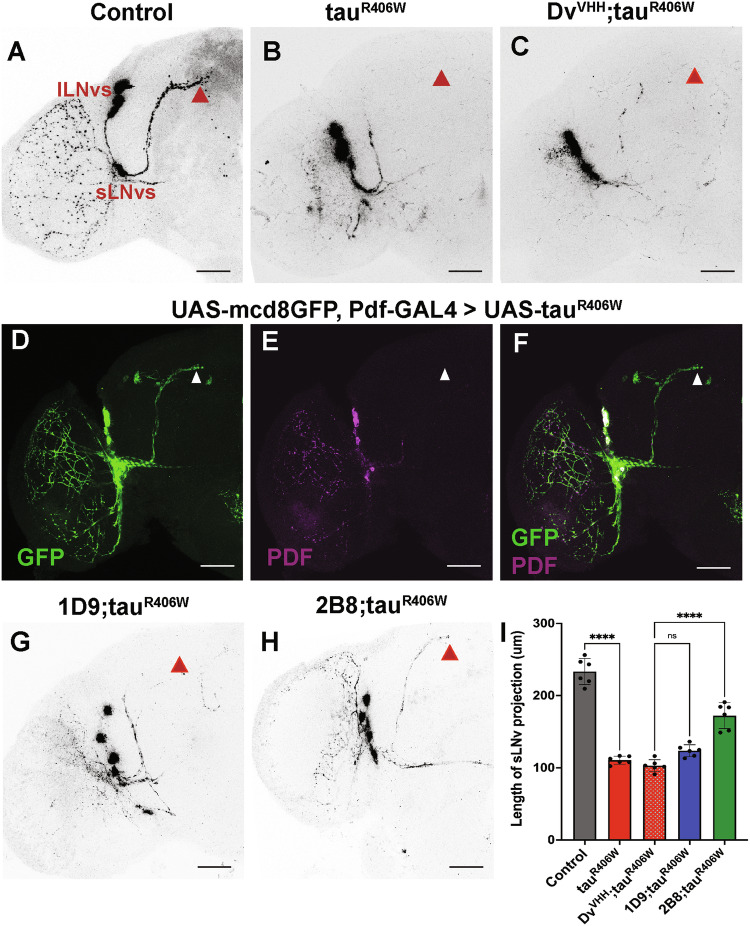
sdAb 2B8 prevents the loss of PDF signal in sLNv axonal projections in the tauopathy model. **A**–**E** Representative images of half of the whole mount *Drosophila* brain. **A** A control without tauR406W expression. Genotype, *Pdf-GAL4/Y*. Highlighted are cell bodies of lLNvs and sLNvs along with sLNv axons projecting to the dorsal protocerebrum (red triangle). **B** Expressing tauR406W only in the Pdf neurons. Genotype, *Pdf-GAL4/Y;; UAS-tauR406W/+*. Intense anti-PDF signal is detected in cell bodies, but not in the sLNv axonal projection. **C** Expressing a control sdAb together with tauR406W in Pdf neurons results in a similar phenotype to (**B**). Genotype, *elav-GAL4/Y; UAS-Dv*^*VHH*^*/+; UAS-tauR406W/+*. **D**–**F** Outlining the cell morphology with GFP in tauR406W-expressing PDF neurons shows intact dorsal projections with sLNv axons projecting to the dorsal protocerebrum (white arrow) but absence of PDF staining in those axons. Genotype *UAS-mcd8gfp/Y; Pdf-GAL4/*+*; UAS-tauR406W*. **G** 1D9 co-expression does not rescue the tauR406W-induced phenotype in the Pdf neurons. Genotype, *Pdf-GAL4/Y; UAS-1D9/*+*; UAS-tauR406W/+*. **H** 2B8 co-expression restores the PDF distribution defect along the sLNv axon dorsal projection in tauR406W expressing neurons. Genotype, *Pdf-GAL4/Y; UAS-2B8/*+*; UAS-tauR406W/+*. The scale bar indicates 20 μm. **I** The quantified length of sLNv projections stained with anti-PDF shows a significant increase in length when 2B8 is expressed. Error bars show SD. *n* = 8 for each genotype, and statistical significance was calculated using ordinary one-way ANOVA (overall *p* < 0.0001, Dunnet’s post-hoc test, ****p < 0.0001).

To examine the impact of 2B8 against tauR406W at the subcellular level, we examined ventral lateral circadian neurons (LNvs) in the *Drosophila* brain. A subset of neurons per brain hemisphere expresses the neuropeptide pigment-dispersing factor (PDF) that regulates the circadian rhythm in *Drosophila* [40, 41]. Specifically, four large PDF-expressing LNvs (lLNvs) per hemisphere send their projections contralaterally and to the optic lobe, and four small LNvs (sLNvs) send their axonal projections dorsally (Fig. 7A). Of note, a recent study reported that the expression of a mutant tau allele (tauE14) in the fly brain causes loss of PDF in the axons [42]. We tested the effect of tauR406W in these neurons by driving tauR406W with the Pdf-Gal4 driver (Fig. 7B). These conditions did not abolish PDF staining, and there was no obvious decrease in signal in the cell bodies. However, the anti-PDF signal was weaker in the sLNv projections, failing to extend into the dorsal protocerebrum (*p* < 0.0001). The anti-PDF labeling along the lLNv optic lobe projections was also lost. Similar results were observed in flies co-expressing tauR406W and control sdAb (Fig. 7C). Next, we co-expressed tauR406W and membrane GFP using Pdf-Gal4. Despite the partial loss of PDF signal in sLNv dorsal projections, GFP labeling demonstrated that the sLNv projections were still present. Yet, PDF mostly remained in the cell bodies, indicating impaired neuronal distribution (Fig. 7D–F). Expressing 1D9 did not completely rescue the PDF distribution pattern along the length of the dorsal projections (*p* = 0.061), showing some misrouting of the axonal projections (Fig. 7G). However, expressing sdAb 2B8 significantly rescued the PDF distribution along the sLNv axonal length (*p* < 0.0001) (Fig. 7H). The optic lobe projections showed anti-PDF signal, although weak, that was absent in tauR406W brains. The quantification of the axonal length of sLNv projection stained with anti-PDF is shown in Fig. 7I. Taken together these data indicate that tauR406W strongly reduces PDF neuropeptide distribution in the sLNv dorsal projections, thus indicating disrupted axonal distribution of the neuropeptide PDF in sLNv neurons. The sdAb 2B8 rescues this tauR406W phenotype in these neurons.

## Discussion

Our findings indicate that transgenic expression of anti-tau single-domain antibodies (sdAbs) has therapeutic potential in the well-established *Drosophila* model of tauopathy. Among the four different sdAbs examined, one was clearly superior, two had moderate efficacy, and one was ineffective. These differences were attributed at least in part to their different binding epitope on the tau protein. The effective sdAbs to a different degree extended the lifespan of the tauopathy flies, cleared pathological tau, and attenuated or blocked tau induced: (1) eye abnormalities; (2) loss of neurons important for memory; (3) axonal protein distribution defects; and (4) locomotor dysfunction.

The transgenic anti-tau sdAbs exhibit no toxicity when expressed independently (Fig. 1). This is promising for their potential application as gene therapy to treat tauopathies. To test their efficacy, we used a tauopathy model that has previously been shown to exhibit neurodegeneration and shortened lifespan [16]. When co-expressed neuronally with familial mutant tauR406W, the 2B8 sdAb strongly improved survival, while two other sdAbs modestly improved the survival of flies expressing the tauR406W mutation (Fig. 1).

The efficacy of the sdAbs was further corroborated with a climbing assay as a behavioral measure of neuronal dysfunction. Expression of tauR406W caused climbing locomotor defects, which became more pronounced in older flies. While all four sdAbs suppressed the climbing defects during the first two weeks, only 1D9 and 2B8 were effective on Day 28, with 2B8 expressing flies having their locomotor ability closest to the normal control group (Fig. 2).

The efficacy differences between sdAbs may at least in part be attributed to their varying binding epitopes and affinity for tau. Although all four sdAbs bind within the microtubule-binding repeat region of 2N4R tau protein, we found that they differ in their binding affinity to tau epitopes within this region both in solid and solution phases (Fig. 4). This region of tau is well established to be involved in tau aggregation [43–45], and is a target of some of the tau antibodies in clinical trials [17]. The most efficacious sdAb, 2B8, binds strongly to tau 271–295 and moderately to tau 307–331 in the solid phase, as well as strongly to positive control PHF-enriched tau from human tauopathy brain. In the solution phase, it binds with high affinity to these two tau regions. The less effective 1D9 sdAb has a similar binding profile to 2B8 in these experiments with a bit less binding to 307–331 in the solid and solution phase. In contrast, the other less effective sdAb, 1F12, binds strongly to tau 307–331 in the solid phase but less well to tau 271–295 or PHF tau in this phase. In the solution phase, 1F12 binds to tau 271–295 with substantially lower affinity than 2B8 or 1D9 and does not recognize tau 307–331. On the other hand, the ineffective 2F12 binds strongly to tau 271–295 in the solid phase but less well to tau 307–331 and PHF. In the solution phase, 2F12 does not recognize tau 271–295 but binds with high affinity to tau 307–331. Taken together, solid phase binding to tau epitopes does not appear to relate well to efficacy. Instead, moderate to strong binding to tau 271–295 in solution appears to be required for efficacy, which is further improved by strong binding to tau 307–331 in solution.

In general, the precise mechanisms underlying the efficacy differences among sdAbs are difficult to decipher. The work of us and others over the years on tau antibody therapies indicates that subtle binding differences of antibodies, even within the same epitope, can greatly affect their efficacy. There is no a priori in vitro way to predict their efficacies in culture or in vivo, and the mechanisms are likely to be multifactorial. For example, high affinity for a particular epitope is not necessarily a prerequisite for efficacy [46]. As for the sdAbs in this study, this may involve their folding within the cell and interaction with tau proteins in the proteasomal and lysosomal clearance pathways which will be explored in future studies.

The results of the survival assay are supported by western blot analysis of tau levels in the fly heads (Fig. 3). Of the four sdAbs, neuronal expression of the 2B8 sdAb, which extended the lifespan of the tauR406W flies close to that of normal flies, led to the lowest tau and phospho-tau levels in the tauopathy flies. The three effective sdAbs, 2B8, 1D9, and 1F12, all reduced these levels in 5-day-old flies, whereas only 2B8 reduced phospho-tau levels in 30-day-old flies. In contrast, the ineffective 2F12 sdAb did not reduce tau or phospho-tau levels at either age.

The *Drosophila* eye has been used by several groups to identify genetic modifiers of tau toxicity. It is a powerful model for understanding pathways that direct cell fate [47]. Developmental expression of human mutant tau has been shown to result in neurodegeneration of the adult *Drosophila* eye [16]. Of the four sdAbs, only 2B8 rescued degeneration of the photoreceptors when expressed pan-neuronally (Fig. 5). These results align with 2B8 being more effective in prolonging the lifespan of flies and clearing pathological tau in their brains. Thus, eye morphology could be used as a quick screening tool to assess the therapeutic potential of sdAbs. For example, we have identified over 50 sdAbs with unique complementarity determining regions (CDRs) from a phage display library derived from a llama that we immunized with tau immunogens [22]. We split these into families based on the binding profile of their cell culture supernatants to various tau immunogens but have only examined a few of those in detail. By transgenically expressing them individually in tauopathy flies, we could relatively quickly identify the most promising ones by examining their eyes.

The mushroom bodies (MBs) play a crucial role in olfactory learning and memory. However, tau-induced degeneration of these neurons compromises this important function [13, 48]. Here, we only examined two of the tau sdAbs, 2B8, and 1D9 (Fig. 6), which had the most functional benefits in the climbing assay, with 2B8 being superior (Fig. 2). Like in the other assays, 2B8 significantly suppressed tauR406W-induced damage to the MBs, whereas 1D9 and a control sdAb were ineffective. Specifically, 2B8 was partially effective when expressed pan-neuronally but completely blocked MB degeneration when expressed via an MB-specific GAL4. Again, these findings bode well for the therapeutic potential of 2B8, in particular as a gene therapy, in which its expression could at least in theory be directed to the most vulnerable brain regions affected by tau pathology.

The role of axonal microtubules in the anterograde transport of synaptic vesicles is crucial for proper neuronal function [49], and deficits in axonal transport are well-established in tauopathies [50]. To examine if 2B8 and 1D9 could rescue these tau-induced deficits, we expressed tau with or without the sdAbs in neurons that express PDF, a neuropeptide that regulates the output of circadian behavioral rhythms. Notably, circadian rhythm is dysregulated in tauopathies [42]. Again, 2B8 significantly improved axonal protein distribution, as evidenced by analyzing the PDF neuronal projections in the flies (Fig. 7), whereas 1D9 and control sdAb were ineffective.

We previously reported that neuronally expressed anti-tau scFv prevents tauopathy-induced phenotypes in *Drosophila* models [18]. It is notable that in that prior report, we showed extensive neurotoxicity in the tauopathy flies as measured by loss of Elav levels on western blots, whereas here Elav levels did not differ between control and tauopathy flies, although the survival profile of the two groups were comparable in both studies. A likely explanation is that in the prior report, protein levels were not normalized before loading the crushed head samples onto the gels, whereas here they were. The heads of the tauopathy flies appear smaller than in control flies resulting in lower protein levels for the same volume of homogenization solution. Therefore, by normalizing the protein levels before loading the samples onto the gel, we are masking the loss of neurons in the tauopathy flies. Regardless, the antibody-mediated clearance of tau in the two studies can be compared, since in the prior study tau levels were normalized to Elav levels. In both studies, the extended lifespan was associated with tau clearance in young flies examined at ages prior to tau-induced death.

Only a few anti-tau sdAbs have been reported previously [21, 22, 51, 52]. One was shown by two-photon imaging via a skull opening to target tau aggregates in two tauopathy mice [52]. Another one from a synthetic phage display library showed some efficacy after its direct brain injection in a lentivirus [51]. In addition, we demonstrated that 2B8 and 1D9 cleared pathological tau prevented tau toxicity in culture, and reduced tau levels in brain interstitial fluid in tauopathy mice as assessed by microdialysis [21]. Subsequently, we reported on the diagnostic imaging potential of 2B8, showing that it allowed non-invasive and specific imaging of tau pathology in mice following its intravenous injection, with the brain signal correlating strongly with lesion burden [22]. Notably, here we show for the first time the therapeutic efficacy of these sdAbs in vivo.

In summary, our findings demonstrate the in vivo molecular, cellular, behavioral and survival effects of anti-tau sdAbs in minimizing or preventing the development of various tau-induced phenotypes in *Drosophila* tauopathy models. Of the four sdAbs we examined, 2B8 was the most effective, increasing survival rate, clearing tau and phospho-tau, and attenuating or preventing tau-induced axonal protein distribution defects, neurodegeneration, and functional deficits. The use of *Drosophila* as a model allows for further investigation into the underlying mechanisms of this therapeutic approach and assessment of its efficacy in other cell types. In particular, transgenic expression of sdAbs in *Drosophila* can guide sdAb-focused gene therapies in mammalian models and hopefully eventually in humans.

### Supplementary information

Supplemental Figures

## Data Availability

All data needed to evaluate the conclusions in the paper are present in the paper and/or the Supplemental Materials.
