# Supplementary material for: Anti-tau single domain antibodies clear pathological tau and attenuate its toxicity and related functional defects
Source: Cell Death Dis. 2024 Jul 30;15(7):543. doi: 10.1038/s41419-024-06927-9 (PMC11289317; doi:10.1038/s41419-024-06927-9)
Supplement: Supplementary file 1 — Supplemental Figures [file 41419_2024_6927_MOESM1_ESM.pdf]

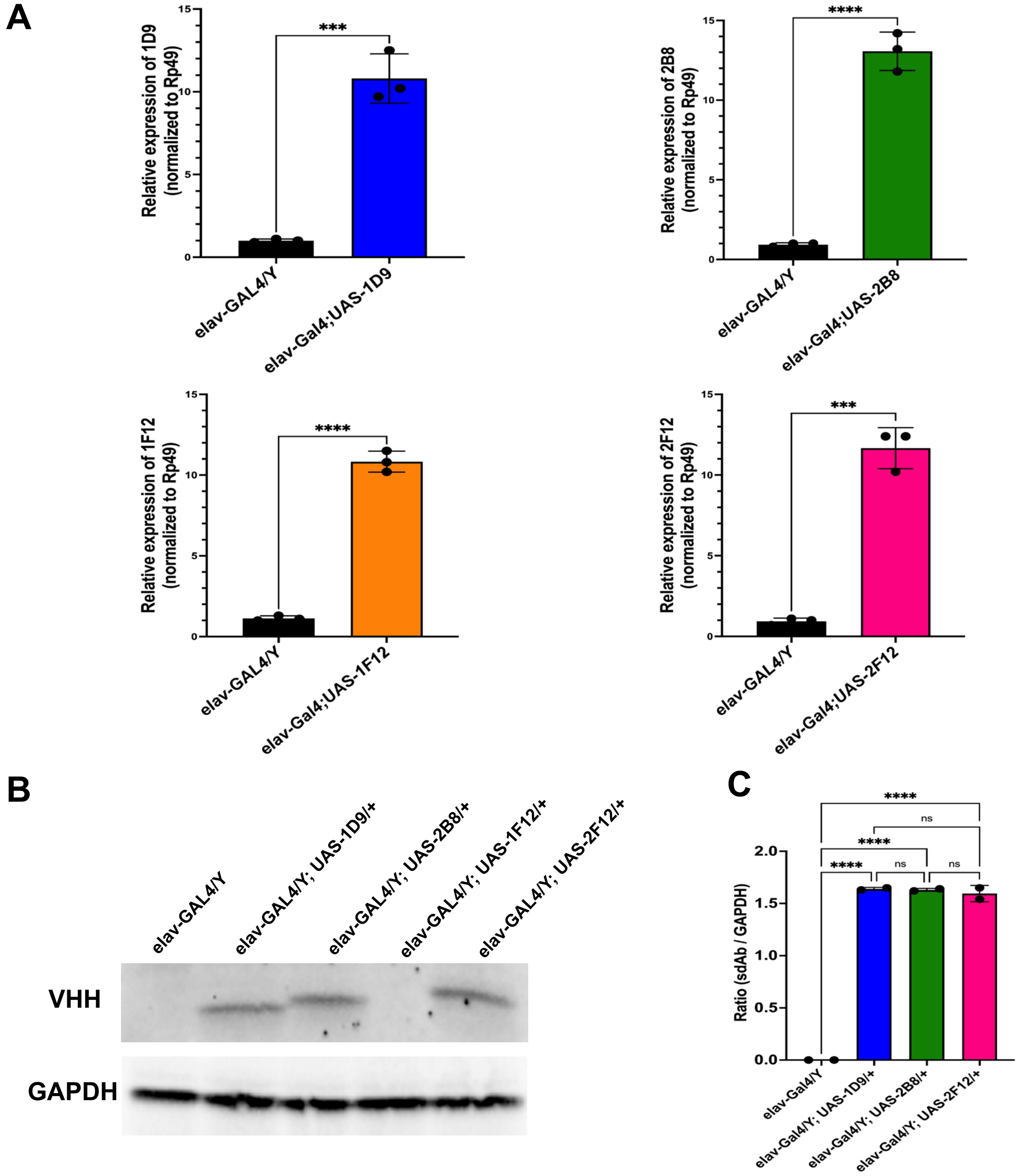

Supplemental Figure 1: Expression of anti-tau sdAbs.

q-RT-PCR of 5 day-old adult fly heads. The transcripts of all the sdAbs (1D9, 2B8, 1F12 and 2F12) are highly induced when expressed pan-neuronally using elav-GAL4 (A). Relative expression was normalized to the expression of housekeeping gene Rp49. Bars represent the average ± SD (Unpaired t-test, two-tailed, *** $p \leq 0.001$, **** $p < 0.0001$) of three biological sets with three technical repeats in each biological set. (B) Representative western blots from 5-day old fly head extracts reacted with anti-VHH antibody and antibody for GAPDH. (C) Quantification of VHH immunoreactive bands normalized to GAPDH levels and plotted as a ratio. Protein was extracted from 25 fly heads for each biological replicate and each bar represents the average of two blots from two biological replicates with error bars indicating standard deviation (SD). Bars represent average ± SD (One-way ANOVA, Tukey's post-hoc test, * $p \leq 0.05$, ** $p \leq 0.01$, *** $p \leq 0.001$, **** $p < 0.0001$). Complete blots for (B) are shown in Supplemental Figure 5).

Immunoblots for sdAb expression related to Supplemental Figure 1

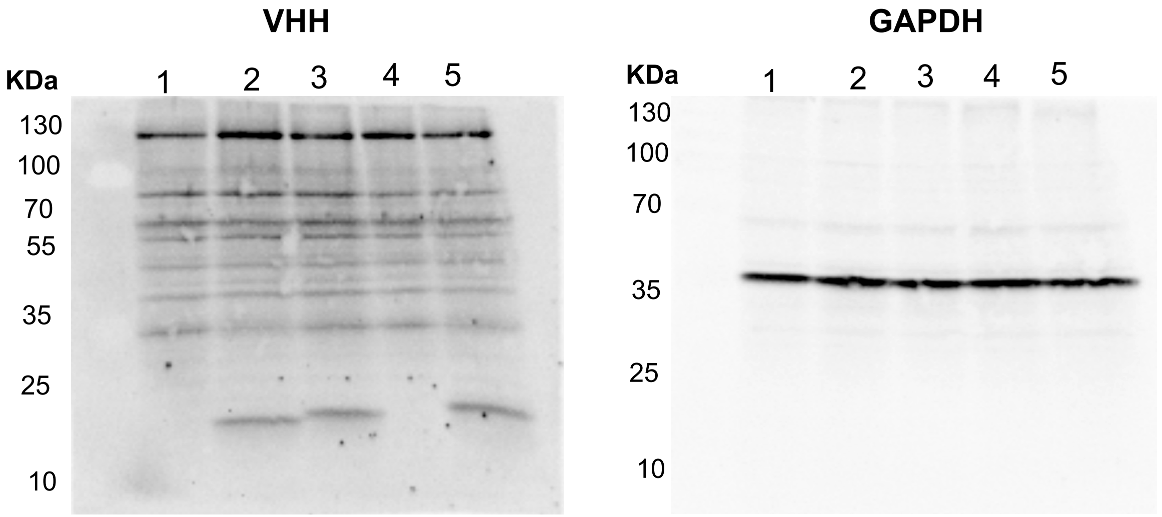

Lane 1: elav-GAL4/Y
Lane 2: elav-GAL4/Y; UAS-1D9/+
Lane 3: elav-GAL4/Y; UAS-2B8/+
Lane 4: elav-GAL4/Y; UAS-1F12/+
Lane 5: elav-GAL4/Y; UAS-2F12/+

Supplemental Figure 2: Complete immunoblots related to Supplemental Figure 1B.
Immunoblots for 5-day-old adult heads for anti-VHH and GAPDH.

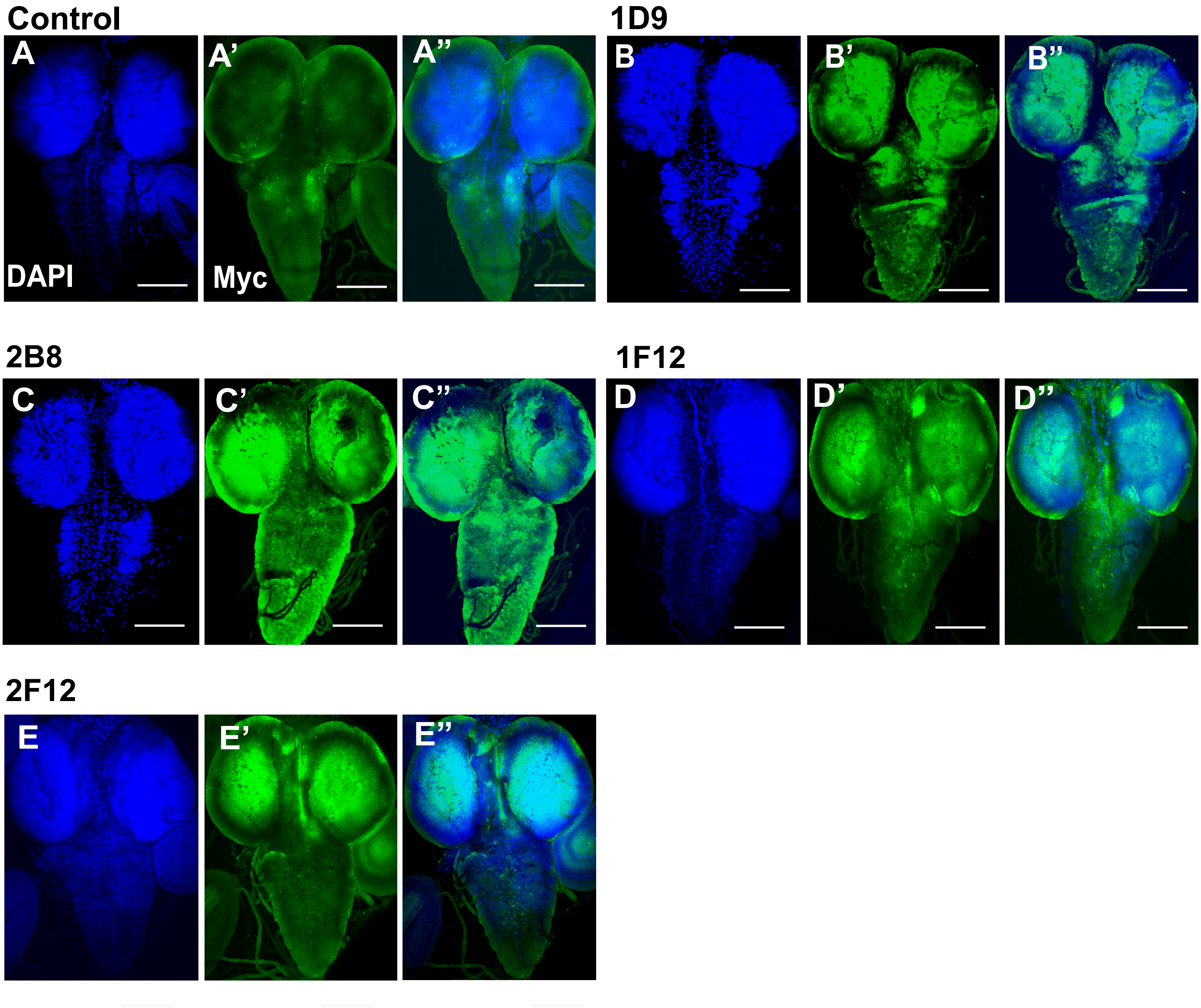

Supplemental Figure 3: Immunohistochemistry depicting expression of anti-tau sdAbs.
Anti-myc labeling of sdAbs in whole mount larval brain. (A-E) DAPI staining (A'-E') anti-myc staining and merged images (A"-E"). DAPI staining (a'-e') anti-myc staining and merged images (a"-e"). n=5 for each genotype. Genotypes 1. elav-GAL4/Y (A-A'') 2. elav-GAL4/Y; UAS-1D9/+ (B-B") 3. elav-GAL4/Y; UAS-2B8/+ (C-C") 4. elav-GAL4/Y; UAS-1F12/+ (D-D") 5. elav-GAL4/Y; UAS-2F12/+ (E-E"). The scale bar represents 100 µm.

Immunoblots for Day 5 related to Figure 3

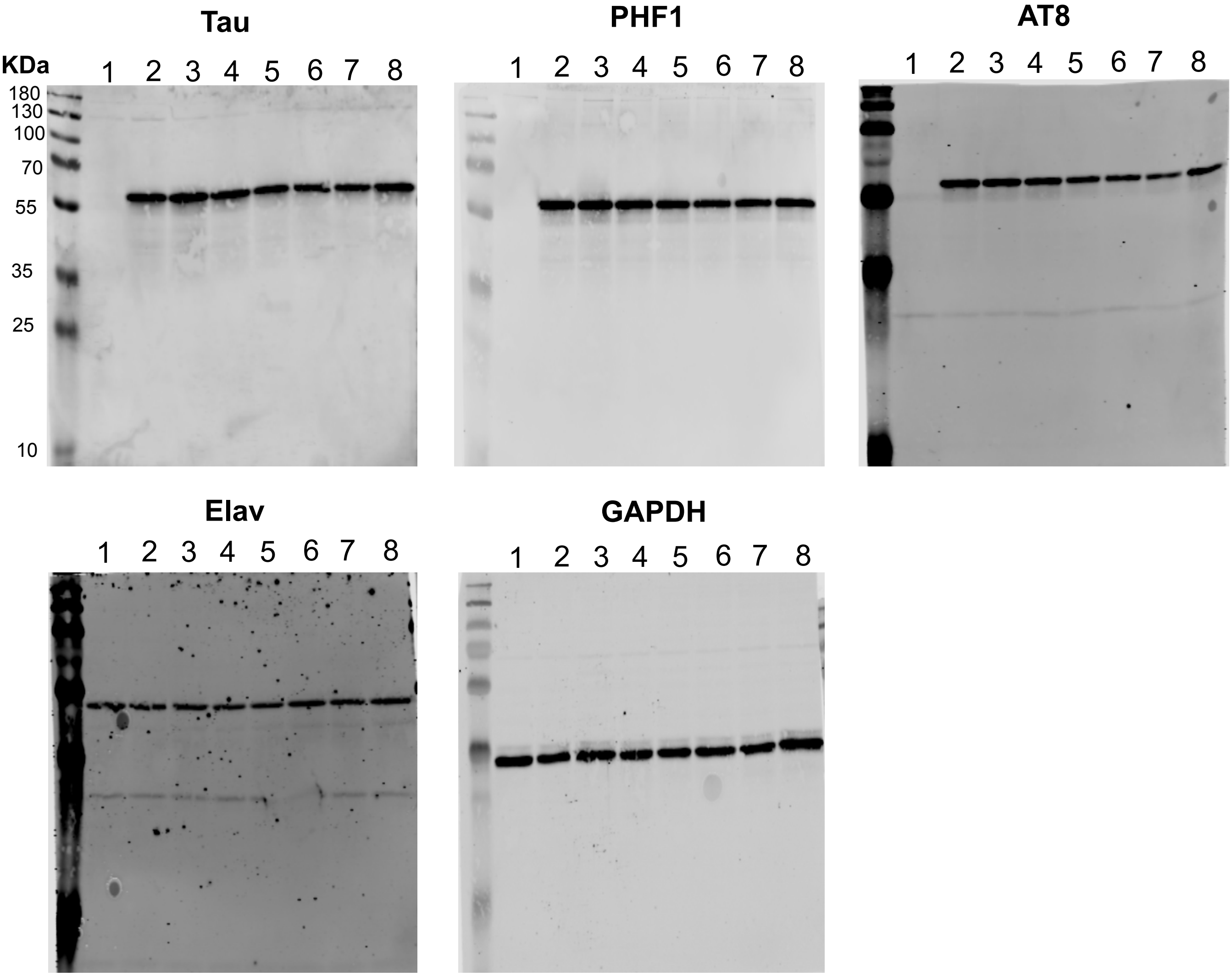

Lane 1: elav-GAL4/Y
Lane 2: elav-GAL4/Y;;UAS-tauR406W/+
Lane 3: elav-GAL4/Y; UAS-EGFP/+; UAS-tauR406W/+
Lane 4: elav-GAL4/Y; UAS-DvVHH/+; UAS-tauR406W/+
Lane 5: elav-GAL4/Y; UAS-1D9/+; UAS-tauR406W/+
Lane 6: elav-GAL4/Y; UAS-2B8/+; UAS-tauR406W/+
Lane 7: elav-GAL4/Y; UAS-1F12/+; UAS-tauR406W/+
Lane 8: elav-GAL4/Y; UAS-2F12/+; UAS-tauR406W/+

Supplemental Figure 4: Complete immunoblots related to Figure 3A-D.
Immunoblots for 5-day-old adult heads for Tau 5A6, PHF1, AT8, Elav, and GAPDH.

Immunoblots for Day 30 related to Figure 3

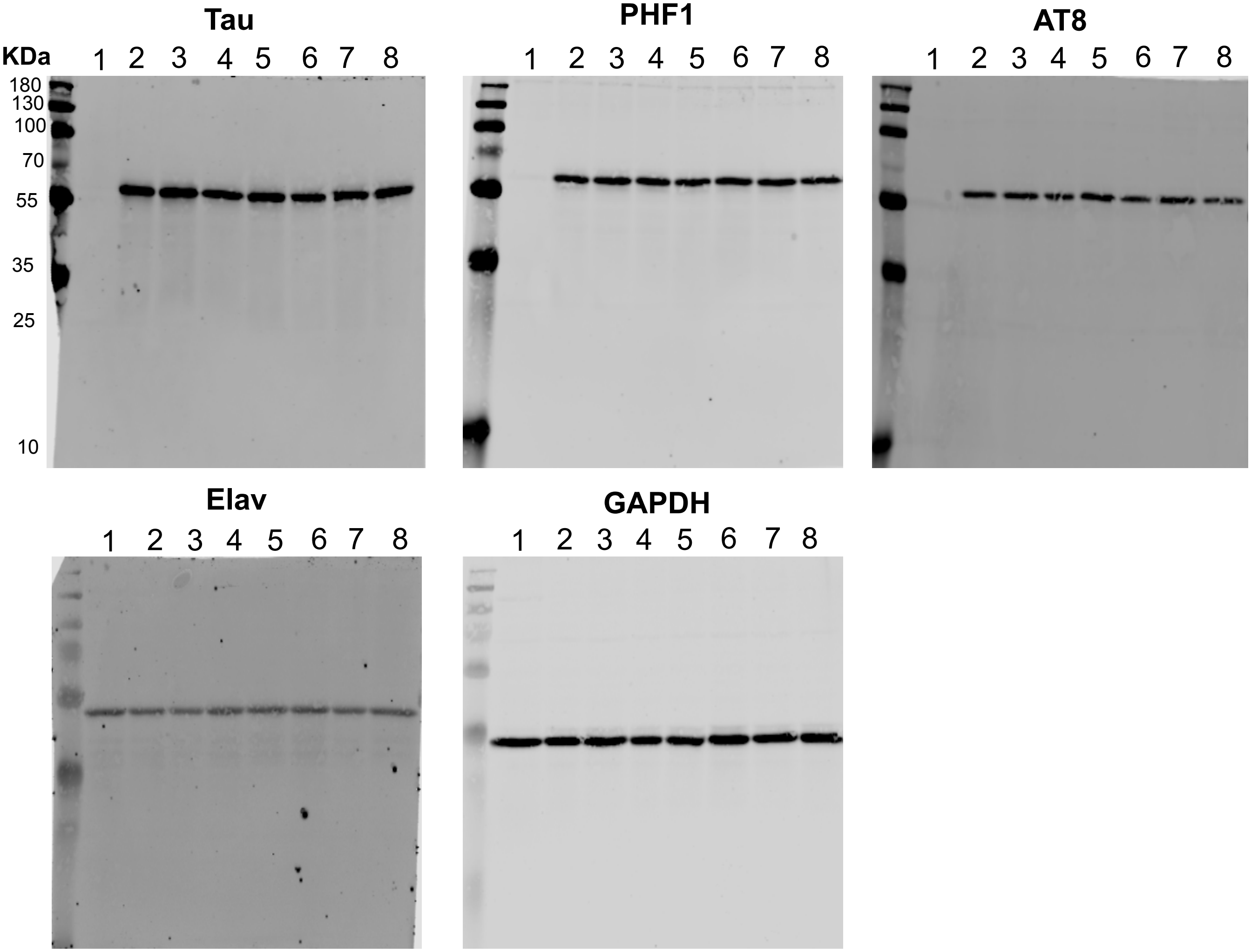

Lane 1: elav-GAL4/Y
Lane 2: elav-GAL4/Y;;UAS-tauR406W/+
Lane 3: elav-GAL4/Y; UAS-EGFP/+; UAS-tauR406W/+
Lane 4: elav-GAL4/Y; UAS-DvVHH/+; UAS-tauR406W/+
Lane 5: elav-GAL4/Y; UAS-1D9/+; UAS-tauR406W/+
Lane 6: elav-GAL4/Y; UAS-2B8/+; UAS-tauR406W/+
Lane 7: elav-GAL4/Y; UAS-1F12/+; UAS-tauR406W/+
Lane 8: elav-GAL4/Y; UAS-2F12/+; UAS-tauR406W/+

Supplemental Figure 5: Complete immunoblots related to Figure 3E-H.
Immunoblots for 30-day-old adult heads for Tau 5A6, PHF1, AT8, Elav, and GAPDH.

A **Epitope mapping of sdAb 2B8**

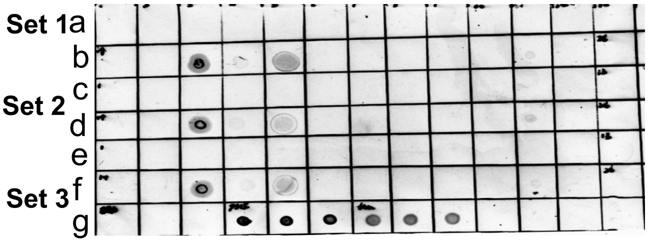

B **Epitope mapping of sdAb 1D9**

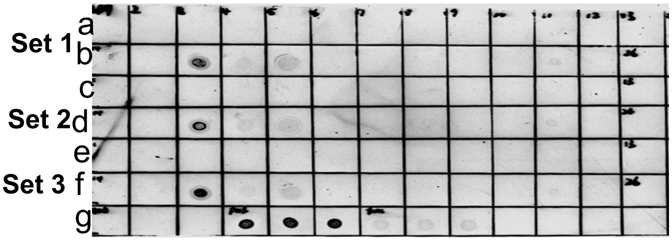

C

sdAb	2B8 and 1D9								
Number	a1, c1, e1	a2, c2, e2	a3, c3, e3	a4, c4, e4	a5, c5, e5	a6, c6, e6	a7, c7, e7	a8, c8, e8	a9, c9, e9	a10, c10, e10
Antigen	Peptide 1	Peptide 2	Peptide 3	Peptide 4	Peptide 5	Peptide 6	Peptide 7	Peptide 8	Peptide 9	Peptide 10
Number	a11, c11,e11	a12, c12, e12	a13, c13, e13	b1, d1, f1	b2, d2, f2	b3, d3, f3	b4, d4, f4	b5, d5, f5	b6, d6, f6	b7, d7, f7
Antigen	Peptide 11	Peptide 12	Peptide 13	Peptide 14	Peptide 15	Peptide 16	Peptide 17	Peptide 18	Peptide 19	Peptide 20
Number	b8, d8, f8	b9, d9, f9	b10, d10, f10	b11, d11, f11	b12, d12, f12	g1-3	g4-6	g7-9		
Antigen	Peptide 21	Peptide 22	Peptide 23	Peptide 24	Peptide 25	BSA	PHF	p-tau		

D **Epitope mapping of sdAb 1F12**

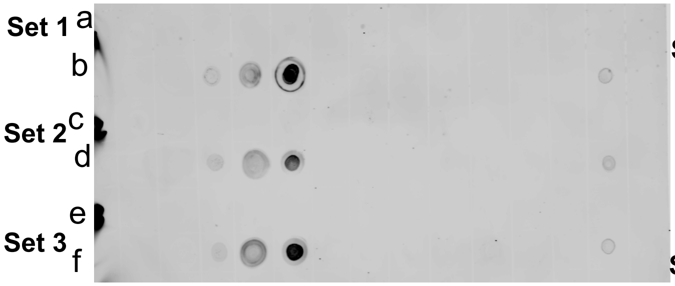

E **Epitope mapping of sdAb 2F12**

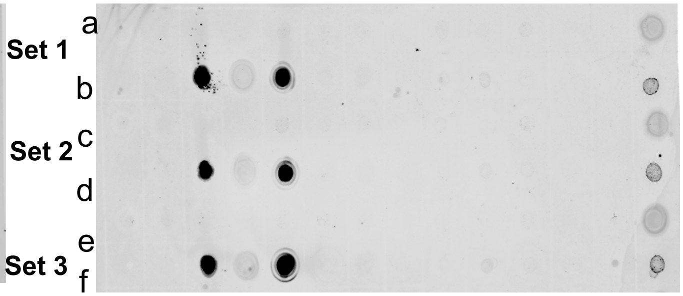

F

sdAb	1F12								
Number	a1, c1, e1	a2, c2, e2	a3, c3, e3	a4, c4, e4	a5, c5, e5	a6, c6, e6	a7, c7, e7	a8, c8, e8	a9, c9, e9	a10, c10, e10
Antigen	Peptide 1	Peptide 2	Peptide 3	Peptide 4	Peptide 5	Peptide 6	Peptide 7	Peptide 8	Peptide 9	Peptide 10
Number	a11, c11,e11	a12, c12, e12	a13, c13, e13	b1, d1, f1	b2, d2, f2	b3, d3, f3	b4, d4, f4	b5, d5, f5	b6, d6, f6	b7, d7, f7
Antigen	Peptide 11	Peptide 12	Peptide 13	Peptide 14	Peptide 15	Peptide 16	Peptide 17	Peptide 18	Peptide 19	Peptide 20
Number	b8, d8, f8	b9, d9, f9	b10, d10, f10	b11, d11, f11	b12, d12, f12	b14, d14, f14	b13, d13, f13			
Antigen	Peptide 21	Peptide 22	Peptide 23	Peptide 24	Peptide 25	BSA	PHF			

G

sdAb	2F12								
Number	a1, c1, e1	a2, c2, e2	a3, c3, e3	a4, c4, e4	a5, c5, e5	a6, c6, e6	a7, c7, e7	a8, c8, e8	a9, c9, e9	a10, c10, e10
Antigen	Peptide 1	Peptide 2	Peptide 3	Peptide 4	Peptide 5	Peptide 6	Peptide 7	Peptide 8	Peptide 9	Peptide 10
Number	a11, c11,e11	a12, c12, e12	a13, c13, e13	b1, d1, f1	b2, d2, f2	b3, d3, f3	b4, d4, f4	b5, d5, f5	b6, d6, f6	b7, d7, f7
Antigen	Peptide 11	Peptide 12	Peptide 13	Peptide 14	Peptide 15	Peptide 16	Peptide 17	Peptide 18	Peptide 19	Peptide 20
Number	b8, d8, f8	b9, d9, f9	b10, d10, f10	b11, d11, f11	b12, d12, f12	b13, d13, f13	b14, d14, f14	a14, c14, e14		
Antigen	Peptide 21	Peptide 22	Peptide 23	Peptide 24	Peptide 25	BSA	PHF	p-tau		

Supplemental Figure 6: Epitope mapping and binding efficacy of anti-tau sdAbs.
Epitope mapping of sdAb 2B8 (A, C), 1D9 (B, C), 1F12 (D, F), and 2F12 (E, G) to tau peptides using dot blot assay in triplicate. Negative control: bovine serum albumin (BSA). Positive control: paired helical filament (PHF)-enriched tau protein from human tauopathy brain.

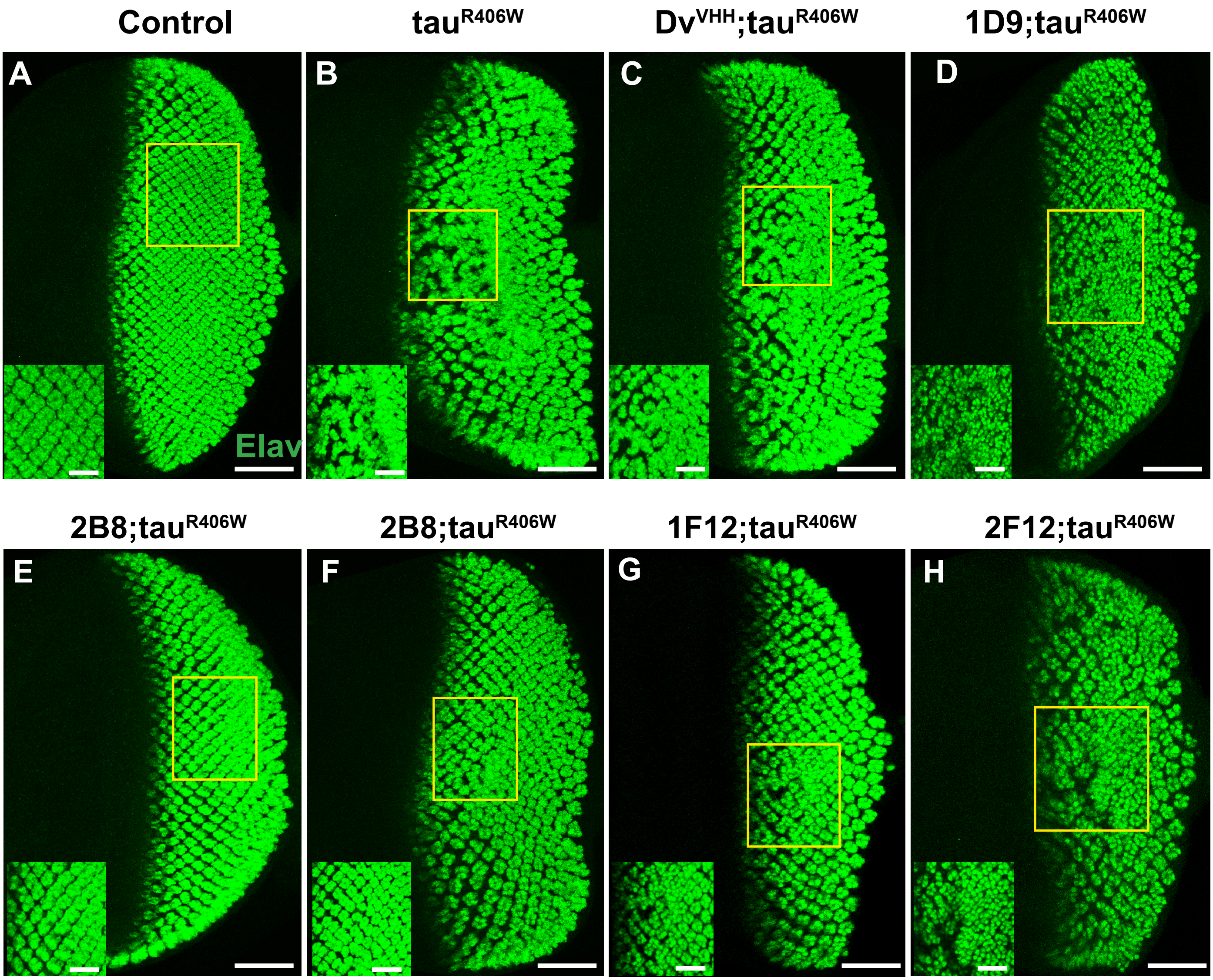

Supplemental Figure 7: sdAb 2B8 prevents abnormal organization of ommatidial cells
Anti-Elav labeling of differentiating photoreceptor neurons in third instar larval eye imaginal disc. Anterior of the eye disc towards left. The inset displays higher magnification images of the yellow highlighted area. Control eye imaginal disc shows properly arranged photoreceptors in (A) elav/Y, whereas in (B-C) the photoreceptor organization is disrupted in tauR406W expressing larval eye imaginal disc. Genotypes: (B) elav-GAL4/Y;; UAS-tauR406W/+. (C) elav-GAL4/Y; UAS-DvVHH/+; UAS-tauR406W/+. (D, G, H) Expressing anti-tau sdAb 1D9, 1F12 and 2F12 did not rescue the tauR406W-induced phenotype. Genotypes: (D) elav-GAL4/Y; UAS-1D9/+; UAS-tauR406W/+. (G) elav-GAL4/Y; UAS-1F12/+; UAS-tauR406W/+ and (H) elav-GAL4/Y; UAS-2F12/+; UAS-tauR406W/+. (E) In contrast, the photoreceptor organization was rescued by expressing 2B8. Genotype: elav-GAL4/Y; UAS-2B8/+; UAS-tauR406W/+. However, in one of the eight eye discs expressing 2B8 and tauR406W, we could see minimal disorganization of photoreceptors (F). The scale bar represents 100 μm. The inset image scale bar is 50 μm.
